# A Comprehensive Review of the Latest Approaches to Managing Hypercholesterolemia: A Comparative Analysis of Conventional and Novel Treatments: Part II

**DOI:** 10.3390/ph18081150

**Published:** 2025-08-01

**Authors:** Narcisa Jianu, Ema-Teodora Nițu, Cristina Merlan, Adina Nour, Simona Buda, Maria Suciu, Silvia Ana Luca, Laura Sbârcea, Minodora Andor, Valentina Buda

**Affiliations:** 1 Faculty of Pharmacy, “Victor Babeș” University of Medicine and Pharmacy, 2 Eftimie Murgu Square, 300041 Timișoara, Romania; narcisa.dinu@umft.ro (N.J.); ema-teodora.nitu@umft.ro (E.-T.N.); sbarcea.laura@umft.ro (L.S.); buda.valentina@umft.ro (V.B.); 2 Doctoral School, “Victor Babeş” University of Medicine and Pharmacy, 2 Eftimie Murgu Square, 300041 Timisoara, Romania; cristina.merlan@umft.ro (C.M.); adina.nour@umft.ro (A.N.); simona.buda@umft.ro (S.B.); 3 Advanced Instrumental Screening Center, Faculty of Pharmacy, “Victor Babeş” University of Medicine and Pharmacy, 2 Eftimie Murgu Square, 300041 Timisoara, Romania; 4 Research Centre for Pharmaco-Toxicological Evaluation, “Victor Babeș” University of Medicine and Pharmacy, 2 Eftimie Murgu Square, 300041 Timisoara, Romania; 5 Faculty of Medicine, “Victor Babeș” University of Medicine and Pharmacy, 1 Eftimie Murgu Square, 300041 Timișoara, Romania; silvia.luca@umft.ro (S.A.L.); andor.minodora@umft.ro (M.A.); 6 Research Centre of Timisoara Institute of Cardiovascular Diseases, “Victor Babeş” University of Medicine and Pharmacy, 300041 Timisoara, Romania; 7 Institute of Cardiovascular Diseases Timisoara, 13A Gheorghe Adam Street, 300310 Timisoara, Romania; 8 Multidisciplinary Heart Research Center, “Victor Babeş” University of Medicine and Pharmacy, 2 Eftimie Murgu Square, 300041 Timisoara, Romania

**Keywords:** hypercholesterolemia, lipid-lowering therapies, cardiovascular risk reduction, PCSK9 inhibitors, inclisiran, bempedoic acid, lomitapide, emerging therapies for dyslipidemia

## Abstract

Cardiovascular disease (CVD) remains the leading cause of mortality worldwide, with hypercholesterolemia identified as a major, but modifiable risk factor. This review serves as the second part of a comprehensive analysis of dyslipidemia management. The first installment laid the groundwork by detailing the key pathophysiological mechanisms of lipid metabolism, the development of atherosclerosis, major complications of hyperlipidemia, and the importance of cardiovascular risk assessment in therapeutic decision-making. It also examined non-pharmacological interventions and conventional therapies, with a detailed focus on statins and ezetimibe. Building upon that foundation, the present article focuses exclusively on emerging pharmacological therapies designed to overcome limitations of standard treatment. It explores the mechanisms, clinical applications, safety profiles, and pharmacogenetic aspects of novel agents such as proprotein convertase subtilisin/kexin type 9 (PCSK9) inhibitors (alirocumab, evolocumab), small interfering RNA (siRNA) therapy (inclisiran), adenosine triphosphate–citrate lyase (ACL) inhibitor (bempedoic acid), microsomal triglyceride transfer protein (MTP) inhibitor (lomitapide), and angiopoietin-like protein 3 (ANGPTL3) inhibitor (evinacumab). These agents offer targeted strategies for patients with high residual cardiovascular risk, familial hypercholesterolemia (FH), or statin intolerance. By integrating the latest advances in precision medicine, this review underscores the expanding therapeutic landscape in dyslipidemia management and the evolving potential for individualized care.

## 1. Introduction

CVD remains the foremost cause of mortality and disability worldwide, imposing a tremendous burden on healthcare systems and individual patients alike [1]. Among the modifiable risk factors driving this epidemic, dyslipidemia—particularly elevated low-density lipoprotein cholesterol (LDL-C)—occupies a central role in the initiation and progression of atherosclerosis, the pathological substrate underlying most cardiovascular events. While widespread statin use over the past two decades has substantially reduced cardiovascular morbidity and mortality, a significant proportion of patients fail to achieve recommended lipid targets or continue to experience residual cardiovascular risk, despite maximal tolerated therapy [2].

This persistent treatment gap has underscored the urgent need for more effective, better-tolerated, and mechanistically diverse lipid-lowering options. In routine clinical practice, managing patients with FH, true statin intolerance, or very high cardiovascular risk presents frequent challenges that often require therapeutic strategies extending beyond conventional statins and ezetimibe. Additionally, atherogenic dyslipidemia—characterized by elevated triglycerides (TGs), low high-density lipoprotein cholesterol (HDL-C), and small dense low-density lipoprotein (LDL) particles—further compounds residual cardiovascular risk in selected patient populations and remains insufficiently addressed by traditional approaches [3].

This review represents the second part of a comprehensive analysis aimed at delineating both established and emerging strategies for hypercholesterolemia management. The first part of this series focused on fundamental pathophysiological processes in lipid metabolism, the formation and clinical consequences of atherosclerotic plaques, cardiovascular risk stratification, and the therapeutic roles of lifestyle modification and foundational pharmacologic therapies such as statins and ezetimibe [4].

Building upon this foundation, Part II of the review explores the evolving landscape of adjunctive and novel pharmacotherapies that are reshaping the modern approach of dyslipidemia management [5,6,7]. Key therapeutic classes examined in this installment include:

- PCSK9 inhibitors, such as alirocumab and evolocumab, monoclonal antibodies that prevent LDL receptor (LDLR) degradation and have demonstrated substantial LDL-C reduction and documented cardiovascular benefits;
- siRNA therapies, exemplified by inclisiran, which leverage ribonucleic acid (RNA) interference to silence *PCSK9* gene expression and provide long-lasting LDL-C lowering with infrequent dosing;
- ACL inhibitors, such as bempedoic acid, which act upstream of 3-hydroxy-3-methylglutaryl coenzyme A (HMG-CoA) reductase, offering LDL-C reduction with a low risk of muscle-related side effects;
- MTP inhibitors, such as lomitapide, which provide an LDLR–independent mechanism for lowering LDL-C in homozygous familial hypercholesterolemia (HoFH);
- ANGPTL3 inhibitors, including evinacumab, which have demonstrated efficacy in reducing LDL-C and TGs, particularly in patients with severely impaired LDLR function.

Each pharmacological class is critically reviewed in terms of molecular mechanisms, pharmacokinetics, clinical indications, safety profiles, drug interaction potential, and relevant pharmacogenetic considerations. Special emphasis is placed on patient subgroups with unique treatment needs, including pediatric, elderly, pregnant, and genetically predisposed populations.

While the introduction of these novel lipid-lowering agents represents a major advance in cardiovascular prevention, their widespread adoption is tempered by considerations of cost-effectiveness and healthcare resource allocation. Therapies such as PCSK9 inhibitors, inclisiran, and evinacumab are associated with substantially higher acquisition costs compared to traditional statins or ezetimibe, raising questions about their economic sustainability, especially in primary prevention settings. Recent pharmacoeconomic analyses have highlighted that these agents can be cost-effective in carefully selected high-risk populations—such as patients with FH, established atherosclerotic cardiovascular disease (ASCVD), or statin intolerance—where their incremental reduction in cardiovascular events translates into meaningful clinical and economic value over the long term. Nevertheless, optimizing the allocation of these therapies requires judicious patient selection guided by risk stratification, shared decision-making, and evolving real-world evidence on clinical and economic outcomes [5,6,7].

By integrating findings from pivotal randomized clinical trials, real-world data, and continuously updated guidelines, this review offers a comprehensive and up-to-date appraisal of the therapeutic armamentarium for dyslipidemia [4]. The adoption of these emerging agents heralds a shift toward more personalized, mechanism-based, and risk-adapted lipid management strategies. As the field continues to advance, clinicians, researchers, and policymakers will need a thorough understanding of these innovations to optimize cardiovascular prevention and improve long-term outcomes across diverse patient populations.

## 2. Pharmacological Treatments

### 2.1. PCSK9 Inhibitors

PCSK9 inhibitors are a modern class of lipid-lowering medications primarily used to manage dyslipidemia and ASCVD. This pharmacological class has been increasingly incorporated into contemporary clinical practice, with its role formally acknowledged in major international guidelines as a cornerstone of adjunctive lipid-lowering therapy in the context of high-risk cardiovascular prevention [3,8,9]. The two main agents currently approved for clinical use are alirocumab and evolocumab. These are fully human monoclonal antibodies that specifically bind to circulating PCSK9, inhibiting its interaction with LDLR [10,11]. This class of inhibitors emerged to address significant clinical needs. Many patients at very high risk of cardiovascular events do not reach their LDL-C targets despite using high-intensity statins and ezetimibe. Additionally, some patients are intolerant to statins or experience adverse effects that prevent their optimal use. PCSK9 inhibitors offer an effective, safe, and well-tolerated solution in these cases, with strong evidence from large randomized trials supporting their cardiovascular benefits [12,13].

#### 2.1.1. Mechanism of Action

**Figure 1:**
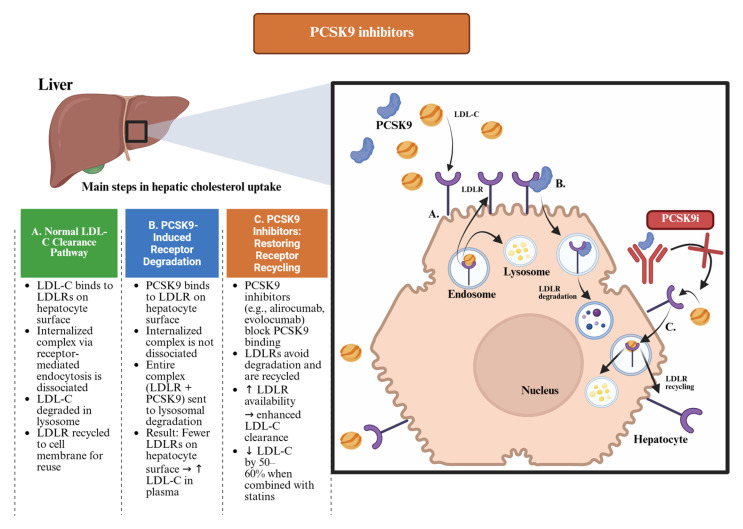
PCSK9 inhibitors—mechanism of action (Created with BioRender.com). Abbreviations: PCSK9—proprotein convertase subtilisin/kexin type 9; LDL-C—low-density lipoprotein cholesterol; LDL-R—low-density lipoprotein receptors; PCSK9i—proprotein convertase subtilisin/kexin type 9 inhibitors; ↑—increase; ↓—decrease; →—which leads to. Legend: A. LDL-C binds to the LDLR and enters the hepatocyte via receptor-mediated endocytosis. From there, the complex (LDLR—LDL-C) enters the lysosome, LDL-C will be degraded, while the LDLR gets recycled, is externalized at the surface of the membrane and the cycle restarts. B. PCSK9 protein binds to LDLR and enters the hepatocyte via receptor-mediated endocytosis. Due to the presence of PCSK9, the endosome can no longer dissociate this complex, so the LDLR is degraded together with PCSK9 protein. This leads to a decrease in the expression of LDLRs on the surface of the hepatocyte. C. PCSK9 inhibitor specifically binds to the PCSK9 protein, thus blocking its binding to the LDLR. Therefore, the LDLR will be recycled by the same mechanism previously described in point A.

The PCSK9 protein is predominantly synthesized by hepatocytes and secreted into the systemic circulation. Under physiological conditions, PCSK9 binds to the LDLR on the surface of liver cells. After internalization of the PCSK9–LDLR complex, PCSK9 promotes lysosomal degradation of the receptor, thereby reducing its availability for recycling to the cell surface. This mechanism results in a reduced number of LDLRs on the surface of hepatocytes and, consequently, in a diminished clearance of LDL-C from the circulation (Figure 1). Monoclonal antibodies, such as alirocumab and evolocumab, inhibit this process by binding to circulating PCSK9, thereby preventing its interaction with the LDLRs (Figure 1) [14].

This preservation of LDLRs enhances receptor recycling and increases the hepatic clearance of LDL-C. As a result, treatment with these agents can reduce LDL-C levels by approximately 50–70%, depending on baseline values and concomitant lipid-lowering therapy (Appendix A, Table A5). This mechanism (Figure 1) represents a paradigm shift in the management of dyslipidemia, in that it aims to prevent LDLR degradation, rather than inhibiting cholesterol synthesis, and has been shown to have a significant clinical impact in reducing cardiovascular risk [11,13].

#### 2.1.2. Clinical Indications

The indications for the use of PCSK9 monoclonal antibodies have been clearly defined by major cardiovascular societies, including the European Society of Cardiology (ESC), the European Atherosclerosis Society (EAS) and the American Heart Association (AHA) [3,9]. These medications are primarily recommended for individuals at high or very high cardiovascular risk who are unable to achieve therapeutic targets for LDL-C, despite using the highest tolerated dose of statins, either alone or in combination with ezetimibe. A key indication for PCSK9 inhibitors is the secondary prevention of ASCVD in patients with a history of myocardial infarction, ischemic stroke, or symptomatic peripheral arterial disease. In this population, failure to achieve LDL-C levels below 55 mg/dL, according to European guidelines, or below 70 mg/dL, according to American recommendations, clearly indicates the need to start therapy with PCSK9 inhibitors [3,9,10,15]. Additionally, individuals withFH, especially those with heterozygous or homozygous mutations affecting LDLR function, are considered suitable candidates for PCSK9 inhibitor treatment. These patients often have significantly elevated LDL-C levels from an early age, and PCSK9 inhibition serves as an effective adjunctive strategy in their lipid-lowering treatment plans.

Another important indication for the use of PCSK9 inhibitors is true statin intolerance, which is defined as a patient’s inability to tolerate at least two different statins due to side effects such as myalgia, fatigue, or elevated creatine kinase levels. In such cases, PCSK9 inhibitors provide an effective and well-tolerated alternative for achieving lipid targets and reducing cardiovascular risk. In certain situations, these agents may be used even in the absence of statin therapy, especially when LDL-C levels are significantly elevated and the patient’s risk of recurrent cardiovascular events is deemed high [12,13].

#### 2.1.3. Safety Profile and Adverse Effects

PCSK9 inhibitors are generally well-tolerated, with a favorable safety profile compared to other lipid-lowering therapies. The most common adverse reactions reported in clinical trials and real-world clinical practice are limited to mild local reactions at the injection site, such as erythema, swelling, or pruritus, which rarely necessitate treatment discontinuation. Systemic side effects are rare and may include nasopharyngitis, flu-like symptoms, and headache. Notably, a low incidence of musculoskeletal symptoms is observed, and PCSK9 inhibitor treatment has not been associated with the myopathy or rhabdomyolysis commonly seen in statin-intolerant patients. In addition, long-term follow-up data of PCSK9 inhibitor treatment have not confirmed concerns about hepatotoxicity, renal impairment, or neurocognitive decline. Unlike high-dose statin therapy, PCSK9 inhibitors have not been associated with an increased risk of new-onset type 2 diabetes and do not appear to negatively impact glycemic control in patients with pre-existing diabetes. These findings support the assertion that PCSK9 inhibitors are not only effective but also safe for long-term use in high-risk populations withCVD. Due to their neutral interaction profile, they can be administered alongside other lipid-lowering agents without the risk of significant pharmacokinetic interference [10,13,16].

#### 2.1.4. Contraindications and Precautions

PCSK9 inhibitors have relatively few contraindications, making them an attractive option for patients with complex or comorbid conditions. The main absolute contraindication is hypersensitivity to the active substance or any of the excipients in the product. Their use during pregnancy and lactation is generally not recommended due to insufficient safety data in these populations. While the pharmacokinetics of these agents are not significantly affected in patients with mild-to-moderate hepatic or renal dysfunction, caution is advised in cases of severe hepatic impairment, given the limited clinical experience (Appendix A, Table A6 and Table A7). The issue of immunogenicity has been studied, revealing that the formation of anti-drug antibodies—though reported in rare cases—has not been linked to a loss of efficacy or anaphylactic reactions. Ongoing monitoring and pharmacovigilance efforts continue to support the favorable tolerability and safety profile of these agents across various patient groups [10,11,12].

#### 2.1.5. Dosage and Administration

Both alirocumab and evolocumab are administered via subcutaneous injection and can be self-injected by patients after receiving proper instructions. Evolocumab offers two treatment regimens: 140 mg every two weeks or 420 mg once a month. Both options demonstrate clinical equivalence in lowering LDL-C levels and providing cardiovascular protection. Alirocumab is typically initiated at a dose of 75 mg every two weeks, with the option to increase to 150 mg every two weeks based on LDL-C levels and therapeutic goals. In certain clinical situations, a monthly dose of 300 mg may also be used. Injections can be given in the abdomen, thigh, or upper arm, and it is recommended to rotate injection sites in order to minimize the risk of local irritation. Both medications are stable at room temperature for a limited time and come in autoinjectors or pre-filled syringes to make administration easier for patients. It is crucial to adhere strictly to the prescribed injection schedule to maintain LDL-C reduction and achieve the best therapeutic outcomes [10,11].

#### 2.1.6. Drug Interactions

Monoclonal antibodies (i.e., alirocumab and evolocumab) are not metabolized by cytochrome enzymes and generally have a low potential for pharmacokinetic drug–drug interactions. However, clinicians should be aware of certain pharmacodynamic implications (Appendix A, Table A1). Because these medications affect lipid metabolism, using them in conjunction with other lipid-lowering agents, especially statins and ezetimibe, may lead to additive effects on LDL-C reduction [17]. These combinations are commonly employed in clinical practice, particularly for patients at high or very high cardiovascular risk. Caution is advised when administering PCSK9 inhibitors alongside agents that affect the immune response, such as corticosteroids or immunosuppressants, due to the theoretical risk of reduced efficacy or an enhanced immune response to the monoclonal antibodies. While combining PCSK9 inhibitors with anticoagulants or antiplatelet agents is not strictly prohibited, it should be approached with caution, as rare bleeding events have been reported in large clinical trials focused on cardiovascular outcomes, although a clear causal relationship has not been established [18]. There is no evidence of significant interactions between PCSK9 inhibitors and oral hypoglycemic agents or antihypertensive drugs, allowing for their safe co-administration. Nevertheless, in a clinical setting, it is crucial to monitor for potential additive effects on hepatic function, particularly when multiple lipid-lowering agents are used together [17,18].

#### 2.1.7. Pharmacogenetic Considerations

Pharmacogenetic variability significantly influences the lipid-lowering response to PCSK9 inhibitors, although its clinical applicability currently remains limited and in an early stage of development [19]. Genetic polymorphisms of the *PCSK9* gene, particularly loss-of-function variants, have been associated with lower baseline LDL-C levels and a potential increased response to treatment with PCSK9 inhibitors (In other words, the lower baseline LDL-cholesterol levels in individuals with mutations that cause loss of function of the *PCSK9* gene are explained by the fact that, in the absence of the PCSK9 protein, LDL receptors are no longer degraded, which leads to an increase in their number on the hepatocytes surface and, implicitly, to a more efficient clearance of LDL-C from the circulation [20]). A subsequent increase in LDL-Cin people with a loss-of-function mutation in the *PCSK9* gene is possible due to the influence of environmental factors (diet, lifestyle), age-related metabolic changes, complex genetic interactions, or liver conditions that can reduce the efficiency of LDLRs, all of which can counteract the initial beneficial effect of the mutation [21].

Conversely, individuals with HoFH due to null mutations in the *LDLR* gene may exhibit an attenuated response, as the therapeutic efficacy of PCSK9 inhibition is contingent upon the presence of functional LDLRs (As the biological effects of PCSK9 and its various gene polymorphisms depend on the presence of at least one functional LDLR, the complete lack of LDLR—found in HoFH patients—deprives PCSK9 of its substrate of action; therefore, inhibition of PCSK9 will not produce clinical consequences, as there is no longer an available target on which to act [22]). Notably, patients harboring *LDLR*-defective mutations may still derive substantial benefit, albeit to a lesser extent than heterozygous familial hypercholesterolemia (HeFH) carriers. In addition, the genetic architecture responsible for elevated lipoprotein(a) (Lp(a)) levels—an independent atherogenic risk factor—may influence the response to PCSK9 inhibitors, with these agents demonstrating modest but clinically relevant reductions in Lp(a) concentrations [10,22].

From a clinical perspective, pharmacogenetic testing is not currently mandatory for prescribing PCSK9 inhibitors. However, clinicians are encouraged to consider genetic testing in patients with severe hypercholesterolemia unresponsive to standard therapies, particularly when HoFH is suspected. In such cases, complete genotyping for mutations in the *LDLR*, apolipoprotein B (*ApoB*), and *PCSK9* genes, along with the use of polygenic risk scores (PRS) to distinguish polygenic hypercholesterolemia, can guide the selection and intensity of therapy. Next-generation sequencing is the most efficient method for this purpose and can be integrated into cascade screening strategies to identify family members at risk [22,23].

#### 2.1.8. Special Populations—Pregnancy, Pediatrics, Elderly

In special populations such as pregnant women, children, and the elderly, the use of PCSK9 inhibitors necessitates individualized risk-benefit assessment. During pregnancy, lipid metabolism undergoes significant physiological modifications, including increases in total cholesterol and LDL-C levels, which are essential for fetal development. Consequently, lipid-lowering therapy is generally avoided during gestation unless the risk of cardiovascular events is deemed imminent. PCSK9 inhibitors, being large monoclonal antibodies, do not cross the placenta during the first trimester, but placental transfer may occur in later stages of gestation. Currently, data on the teratogenic potential and fetal safety of PCSK9 inhibitors are insufficient, which is why their use is not recommended during pregnancy. Also, breastfeeding should be avoided during treatment with PCSK9 inhibitors, as it is not known whether these agents are excreted in breast milk [10,17,18].

In pediatric patients, especially those with genetically confirmed heterozygous or homozygous familial hypercholesterolemia, early initiation and intensification of lipid-lowering therapy are essential to delay the progression of atherosclerosis. Evolocumab has been approved for use in children aged 10 years and older with HoFH or HeFH who require additional LDL-C reduction as an adjunct to statin and ezetimibe therapy. Although the long-term safety and efficacy profile in this group are still being evaluated, the data available to date support its inclusion in the therapeutic options for pediatric FH [10].

Among elderly individuals, the use of PCSK9 inhibitors appears to be both efficacious and well-tolerated. Aging is associated with an increased burden of atherosclerotic disease, and clinical trials have demonstrated consistent LDL-C lowering and cardiovascular event reduction across older age groups. Nevertheless, polypharmacy, frailty, and comorbidities must be carefully considered when initiating therapy. In the context of primary prevention, it is essential that physicians perform an individualized analysis of life expectancy, the likelihood of adherence to treatment, and the clinical risk-benefit ratio of PCSK9 therapy in these elderly patients [10,17].

PCSK9 inhibitors have established themselves as an effective and intensive therapeutic option in the management of hypercholesterolemia, particularly among patients with high or very high cardiovascular risk who fail to achieve LDL-C targets with standard/conventional therapy [24,25]. By inhibiting LDLR degradation, monoclonal antibodies such as alirocumab and evolocumab cause significant and sustained reductions in LDL-C levels, with demonstrated benefits in reducing major adverse cardiovascular events [25]. Their favorable safety profile and additive efficacy, when combined with statins and ezetimibe, have further strengthened their clinical utility. As evidence from long-term studies accumulates, PCSK9 inhibitors continue to redefine the therapeutic landscape of lipid disorders, especially in FH and statin-intolerant populations [25]. Despite the high cost, ongoing cost-effectiveness analyses, along with extensive initiatives to improve access, are contributing to the gradual integration of these therapies into the standard care pathways of patients with hypercholesterolemia [26].

Beyond their demonstrated efficacy in LDL-C reduction, the implementation of PCSK9 inhibitors has raised important pharmacoeconomic considerations. Due to their high acquisition costs, their cost-effectiveness has been shown to be most favorable in patients at very high cardiovascular risk, particularly in secondary prevention. Recent initiatives to improve access include value-based pricing agreements, national reimbursement protocols, and patient assistance programs. For example, countries such as the United Kingdom and Germany have adopted risk-sharing schemes and prescribing criteria to optimize cost-effectiveness, while in the United States, both public and private payers have negotiated pricing and launched access programs to expand eligibility [27,28,29,30].

In Romania, however, access to PCSK9 inhibitors remains limited by protracted reimbursement timelines and structural inefficiencies in health technology assessment processes. Recent analyses indicate that conditionally approved medications may experience, on average, delays of up to 274 days longer to reach reimbursement compared to unconditionally approved therapies. This lag poses significant barriers to timely access for high-risk cardiovascular patients. Unlike countries that have implemented managed-entry agreements or value-based reimbursement models, Romania’s access pathways remain underdeveloped, highlighting an unmet need for systematic pharmacoeconomic reform to support equitable implementation of high-cost, high-impact therapies [31].

### 2.2. siRNA Therapies

siRNAs represent an innovative class of gene-silencing therapeutics that modulate gene expression at the post-transcriptional level by activating the endogenous RNA interference (RNAi) pathway [32,33]. In the context of dyslipidemia, siRNA-based therapies have emerged as promising solutions for targeting essential liver genes involved in lipid metabolism. These therapies provide long-lasting lipid-lowering effects with reduced administration requirements. Among these, inclisiran is currently the most advanced and clinically approved siRNA lipid-lowering agent. It is specifically designed to inhibit the hepatic synthesis of PCSK9, a central regulator of LDL-C clearance. Unlike conventional lipid-lowering therapies, such as statins, ezetimibe, and monoclonal antibodies targeting PCSK9, which act at the protein or receptor level, inclisiran works upstream by degrading the messenger RNA of PCSK9. This mechanism blocks protein synthesis by inhibiting protein translation [33]. Inclisiran exemplifies the integration of precision pharmacology with RNA biotechnology. It employs a hepatocyte-targeted delivery system based on N-acetylgalactosamine conjugation (GalNAc), ensuring tissue specificity, increased potency, and sustained long-term efficacy [32,34].

#### 2.2.1. Mechanism of Action

Inclisiran exerts its lipid-lowering effect by harnessing the endogenous RNA interference pathway, specifically reducing the hepatic synthesis of PCSK9. The molecule is a chemically stabilized, double-stranded siRNA conjugated to triantennary N-acetylgalactosamine, which facilitates selective uptake into hepatocytes by binding to the asialoglycoprotein receptor (ASGPR), abundantly expressed on the surface of liver cells. After internalization, inclisiran dissociates, and the antisense strand is incorporated into the RNA-induced silencing complex (RISC). This complex subsequently binds to the complementary mRNA of PCSK9, causing its degradation and blocking translation into functional proteins [35,36].

**Figure 2:**
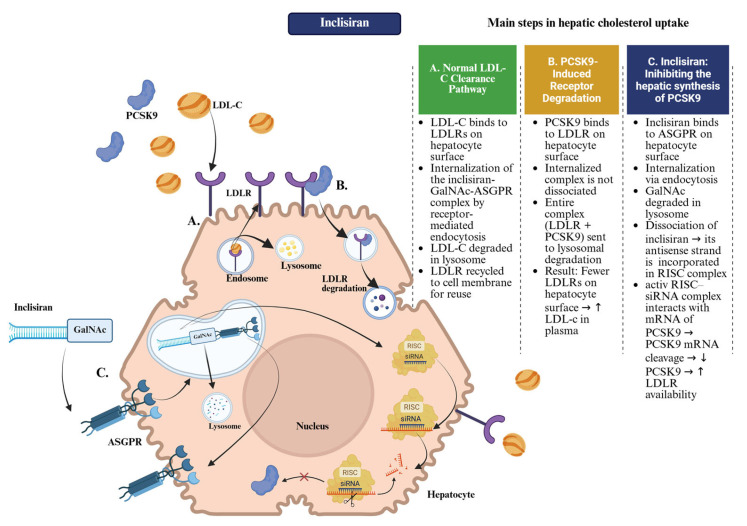
Inclisiran—mechanism of action (Created with BioRender.com). Note: Inclisiran is a chemically synthesized small interfering RNA (siRNA) therapy that targets PCSK9 mRNA in hepatocytes, leading to its degradation via the RNA-induced silencing complex (RISC) and thereby reducing PCSK9 protein synthesis. Post-transcriptional modifications refer to chemical or structural alterations made to RNA molecules after they are transcribed from DNA but before they are translated into proteins. Inclisiran incorporates several such modifications to enhance its stability, efficacy, and liver-specific delivery. These include 2′-O-methyl or 2′-fluoro substitutions on the ribose sugars to protect against nuclease degradation, phosphorothioate backbone linkages for increased enzymatic resistance, and conjugation to triantennary N-acetylgalactosamine (GalNAc), which facilitates selective uptake by hepatocytes via asialoglycoprotein receptors. In contrast, post-translational modifications are chemical changes that occur to proteins after their synthesis (translation), such as glycosylation, phosphorylation, or proteolytic cleavage, which often affect protein function, localization, or stability. While inclisiran itself does not undergo post-translational modifications, its mechanism of action prevents the production of PCSK9 protein, thereby indirectly abolishing any post-translational modifications that PCSK9 would normally experience. This highlights inclisiran’s novel role as a post-transcriptional gene-silencing therapy with implications in lipid lowering and cardiovascular risk reduction [38,39]. Abbreviations: mRNA—messenger ribonucleic acid; siRNA—small interfering ribonucleic acid; PCSK9—proprotein convertase subtilisin/kexin type 9; LDL-C—low-density lipoprotein cholesterol; LDLR—low-density lipoprotein cholesterol receptor; GalNAc—N-acetylgalactosamine; ASGPR—asialoglycoprotein receptor; RISC—ribonucleic acid-induced silencing complex; →—which leads to; ↓—decrease; ↑—increase.

By reducing intracellular PCSK9 levels, inclisiran indirectly increases the number of LDLRs available on the hepatocyte surface. Since PCSK9 promotes the lysosomal degradation of LDLRs, its synthesis inhibition results in increased receptor recycling and enhanced clearance of circulating LDL-C. It is important to note that this mechanism (Figure 2) causes a prolonged effect on LDL-C levels, with reductions sustained over several months after a single administration (Appendix A, Table A5). This long-lasting lipid-lowering effect is mechanistically explained by the extended intracellular activity of the RISC–siRNA complex, which enables continuous degradation of PCSK9 mRNA long after drug administration, thereby ensuring durable suppression of PCSK9 synthesis and sustained LDLR activity [34,37]. Inclisiran’s pharmacodynamic effect is largely independent of plasma concentrations and is instead sustained by the intracellular persistence and turnover of RISC-bound siRNA in hepatocytes. These aspects give inclisiran a distinct/unique pharmacological profile characterized by slow onset of action and long-lasting efficacy, requiring only two maintenance administrations per year, following a loading schedule of two initial doses given at Day 0 and Month 3 [33,36].

#### 2.2.2. Clinical Indications

Inclisiran is approved for the treatment of adults with primary hypercholesterolemia, including HeFH or mixed dyslipidemia. It is indicated as an adjunct to diet. It is frequently used in combination with statins or conjunction with statins and other lipid-lowering therapies in cases where LDL-C goals are not achieved despite the use of maximum tolerated doses. Inclisiran may also be considered in patients who are statin-intolerant or who experience significant adverse effects from other lipid-lowering medications [40].

In the ORION-10 and ORION-11 clinical trials, inclisiran demonstrated consistent and robust reductions in LDL-C levels (~50%) across a wide range of baseline cardiovascular risk profiles regardless of age, sex, ethnicity, or comorbidities. These trials included both patients with established ASCVD and individuals with increased risk factors, such as diabetes mellitus, hypertension, or elevated baseline LDL-C levels [33,34]. Although data on long-term cardiovascular effects are still accumulating, the magnitude of LDL-C reduction achieved with inclisiran is comparable to that seen in studies with statins and monoclonal PCSK9 inhibitors, suggesting, by extrapolation, a significant potential to reduce cardiovascular morbidity and mortality [41].

Current guidelines endorse inclisiran as a second or third-line agent in lipid-lowering regimens, particularly when lifestyle modifications, statins, and ezetimibe fail to achieve LDL-C targets [3,9].

#### 2.2.3. Adverse Effects and Safety Profile

Inclisiran has been shown to be safe and well-tolerated in both clinical trials and real-world practice. Most often, adverse reactions are limited to the injection site and consist of mild-to-moderate pain, erythema, edema, or pruritus. These manifestations are transient, do not accumulate over time, and usually resolve without the need for medical intervention [42].

Systemic side effects are rare. Notably, inclisiran has not been associated with muscle toxicity, hepatotoxicity, or significant changes in glycemic control, adverse effects that are occasionally observed with statins. Elevations in hepatic transaminases are rare during inclisiran therapy and occur with a frequency similar to that observed in the placebo groups. The effect of inclisiran on immunogenicity appears to be minimal, as no relevant development of neutralizing antibodies or hypersensitivity reactions has been reported to date during treatment. Importantly, inclisiran does not cross the blood–brain barrier and has not been associated with adverse neurocognitive effects [40,42].

In addition to the well-established safety outcomes reported in pivotal phase III trials, recent pharmacovigilance data from real-world settings have offered further insights into the tolerability of inclisiran. A detailed analysis of the EudraVigilance database from 2021 to 2023 highlighted a prevalence of non-serious adverse reactions, most of which were related to the injection site [43]. Although signal detection analyses indicated a slightly increased reporting frequency for terms such as myalgia, elevated LDL-C, and therapeutic ineffectiveness, these events remained infrequent and were rarely severe. It is important to emphasize that no new safety concerns emerged regarding liver function, muscle toxicity, or central nervous system effects. These findings are consistent with the pharmacological specificity of inclisiran for hepatocytes and the absence of systemic accumulation [43,44,45]. This favorable safety profile observed during the post-marketing period supports data from controlled clinical trials, further reinforcing inclisiran’s reputation as a well-tolerated option in lipid-lowering therapy [44,45].

Overall, the safety profile of inclisiran remains favorable, and the lack of daily systemic exposure, due to its hepatic-specific delivery mechanism, likely contributes to its excellent tolerability [46].

#### 2.2.4. Contraindications and Precautions

Inclisiran is contraindicated in individuals with a known hypersensitivity to the active substance or any of the excipients in the formulation [42,47]. Caution is advised in patients with severe hepatic impairment (Child-Pugh Class C) (Appendix A, Table A7), as these individuals were underrepresented in clinical trials, the pharmacodynamic response may be unpredictable due to altered hepatic absorption and metabolism, and there are no data available on the use of inclisiran in this patient population [34,42]. Similarly, data on inclisiran use in patients with end-stage renal disease are limited, although no dose adjustments are currently recommended for mild-to-moderate renal dysfunction (Table A6) [40].

Despite the fact that preclinical studies in animals have not shown teratogenic effects, due to the lack of adequate human data, use in pregnancy is not recommended unless the clinical benefit justifies the potential fetal risk. Breastfeeding is not recommended during treatment due to the lack of data on excretion into breast milk and the uncertainty regarding potential effects on the infant. Pediatric use is not currently approved, and further studies are required to assess safety and efficacy in this population [40].

#### 2.2.5. Dosage and Administration

Inclisiran is administered via subcutaneous injection in the abdomen, upper arm, or thigh [41]. The recommended dosing regimen includes an initial dose on Day 0, followed by a second dose at 3 months and subsequent maintenance doses every 6 months [48]. The fixed dose is 284 mg per injection, and no adjustment is necessary based on body weight, age, gender, or mild-to-moderate hepatic or renal impairment [42].

This infrequent dosing regimen offers a significant advantage in clinical practice, contributing substantially to improved adherence and long-term therapeutic persistence. Unlike statins, which require daily administration, or anti-PCSK9 monoclonal antibodies, administered every two weeks or monthly, inclisiran provides sustained LDL-C control with only two maintenance injections per year. The injection is typically administered in a healthcare setting to ensure correct administration and facilitate patient monitoring. The extended dosing interval is possible due to the intracellular persistence of inclisiran in hepatocytes and the prolonged activity of the RISC, which continues to suppress PCSK9 synthesis for several months after administration [33,47].

#### 2.2.6. Drug Interactions

Inclisiran is not metabolized via the cytochrome P450 system and does not engage common hepatic or renal transporter pathways, making clinically significant drug–drug interactions highly unlikely [42,49]. This favorable pharmacokinetic profile is attributed to its targeted hepatic delivery through GalNAcconjugation and its degradation by endogenous nucleases into naturally occurring nucleotides. As such, inclisiran neither induces nor inhibits CYP enzymes and does not act as a substrate or inhibitor of key transporters such as OATP1B1/1B3, BCRP, or P-gp (BCRP—Breast Cancer Resistance Protein, P-gp—P-glycoprotein) [32,50]. Concomitant use with other lipid-lowering therapies such as statins or ezetimibe has not demonstrated any clinically meaningful interaction (Appendix A, Table A2), and co-administration appears to be both safe and synergistic in reducing LDL-C levels [33]. Nonetheless, as with any new class of therapies, post-marketing surveillance is essential to detect any rare or delayed pharmacological interactions [42].

#### 2.2.7. Pharmacogenetic Considerations

Pharmacogenetics is expected to play a lesser role in the clinical response to inclisiran compared to other lipid-lowering agents. Since inclisiran acts through RNA interference, selectively degrading PCSK9 mRNA in hepatocytes, it is unlikely that genetic polymorphisms influencing CYP enzymes or drug transporters will affect its pharmacokinetics or efficacy. However, genetic polymorphisms affecting LDLR expression or PCSK9 function could theoretically modulate the clinical response to inclisiran, although current evidence does not support the need for genotyping prior to initiating therapy. In patients with rare hereditary forms of hypercholesterolemia, particularly those with HoFH, genetic testing is crucial for identifying individuals with inactive or null mutations in the *LDLR* gene, who may respond suboptimally to PCSK9-targeted therapies. Therefore, it is recommended that physicians consider genetic screening for variants in the *LDLR* or *PCSK9* genes in cases of treatment-refractory hypercholesterolemia, although a specific pharmacogenetic test is not currently required prior to initiation of inclisiran therapy [19,51].

Inclisiran exerts its effect by selectively reducing PCSK9 mRNA expression in hepatocytes through the mechanism of RNA interference, a pathway that bypasses classical enzymatic metabolism and conventional drug transport systems. Therefore, genetic polymorphisms affecting CYP enzymes or hepatic transporters, such as SLCO1B1 or ABCG2, are unlikely to influence the pharmacokinetics or therapeutic efficacy of inclisiran [32].

However, genetic variants affecting LDLR functionality may significantly influence the lipid-lowering response to inclisiran. In particular, patients with HoFH harboring null mutations in both LDLR alleles have shown minimal LDL-C reduction despite effective suppression of circulating PCSK9 levels [19,52]. This underscores the mechanistic dependence of inclisiran and other PCSK9-targeted therapies on the presence of functional LDLRs. Therefore, in patients with FH or treatment-resistant forms, genotyping for mutations in the *LDLR* and *PCSK9* genes may have clinical relevance. Although not mandatory before initiating inclisiran therapy, genetic screening may contribute to adjusting therapeutic expectations and optimizing personalized treatment strategies [53].

#### 2.2.8. Special Populations—Pregnancy, Pediatrics, Elderly

The use of inclisiran in special populations remains poorly characterized, mainly due to limited representation in pivotal trials and ethical constraints on conducting randomized controlled trials in these groups. In pregnancy, no adequate data exist regarding the safety of inclisiran. Although preclinical studies have not shown teratogenicity, physiological changes associated with pregnancy may influence the pharmacokinetics and distribution of inclisiran. Physiologically based pharmacokinetic (PBPK) modeling has been proposed as a tool for estimating maternal-fetal distribution of siRNAs; however, these models are currently theoretical and have not yet been validated for clinical application. Until further safety data are available, the use of inclisiran during pregnancy should be avoided unless there are no suitable alternatives and the potential benefits to the mother justify the possible risks to the fetus [54].

In pediatric populations, inclisiran has not yet received regulatory approval. However, given that early intervention in FH may reduce long-term cardiovascular risk, clinical trials are underway to evaluate its safety and efficacy in pediatric patients. Until the results of ongoing studies are published, the inclisiran use in children remains outside the approved indications. It should be limited to exceptional cases under the supervision of a specialist with experience in pediatric dyslipidemias [55].

In elderly patients, the pharmacokinetic profile of inclisiran does not appear to be significantly altered (Appendix A, Table A6 and Table A7). Clinical trials have included a substantial proportion of older adults and demonstrated comparable efficacy and safety across all age groups. Due to its infrequent administration schedule, inclisiran may represent an important advantage for elderly populations, especially those with polypharmacy or difficulties in maintaining therapeutic adherence [32].

Inclisiran represents a paradigm shift in lipid-lowering therapy by introducing RNA interference as a clinically viable mechanism to target dyslipidemia. Its innovative design, involving liver-targeted delivery of siRNA against PCSK9 mRNA, allows inclisiran to provide a potent LDL-C reduction with a single biannual dosing regimen, thereby significantly improving patient adherence. Due to its high specificity, prolonged effect, and minimal drug interaction profile, inclisiran proves to be a particularly suitable option for managing long-term cardiovascular risk. While outcome data are still emerging, the robust lipid-lowering efficacy and favorable tolerability profile position inclisiran as a complementary or alternative strategy to monoclonal PCSK9 inhibitors, especially in populations where adherence, injection frequency, or cost-effectiveness are concerns [56]. The development of the inclisiran also emphasizes the extended potential of RNA therapies in the management of chronic diseases [53].

### 2.3. An Overview of the Divergent Fates of the LDLR: A Tale of Two Ligands—And One Silencer

As previously discussed, the LDLR plays a central role in the regulation of plasma cholesterol by mediating the clearance of LDL-C from circulation. This transmembrane receptor, expressed on the surface of hepatocytes, binds circulating LDL particles and internalizes them through clathrin-mediated endocytosis. However, the receptor’s ultimate fate—whether it is recycled to the cell surface or degraded—depends critically on the identity of its bound ligand [57,58].

Two ligands compete for the LDLR, each dictating a dramatically different outcome: LDL-C, the physiological cargo, promotes receptor recycling, whereas PCSK9, a post-translational modulator, targets it for degradation. This molecular tug-of-war has profound consequences for cholesterol homeostasis—and has become the basis for innovative therapeutic strategies. Chief among them is inclisiran, a siRNA that silences PCSK9 expression at the genetic level [11,51,59].

LDL-C acts as a ligand that preserves LDLR function. It binds to the receptor via specific ligand-binding LA (ligand-binding type A) repeats—primarily R4 to R7—through non-covalent electrostatic interactions mediated by the ApoB-100 protein. Following internalization, the LDLR–LDL complex is transported to acidified endosomes, where the drop in pH triggers a conformational change in the receptor, leading to the dissociation of LDL. The LDL particle is then directed to lysosomes for degradation, while the unbound receptor is efficiently recycled to the cell surface. This recycling process is highly efficient: a single LDLR molecule can mediate hundreds of uptake cycles, supporting sustained clearance of circulating cholesterol [60,61,62].

In contrast, PCSK9 acts as a molecular brake on LDLR recycling. It binds to the epidermal growth factor-like repeat A (EGF-A) domain of LDLR—a site distinct from the LDL-C binding region. Although this interaction is also non-covalent, it is paradoxically stabilized, rather than weakened, by endosomal acidification. Consequently, the PCSK9–LDLR complex fails to dissociate and is instead shuttled to lysosomes for degradation. The biological result is a reduced density of LDLRs on the hepatocyte surface, impairing LDL-C clearance and contributing to elevated plasma cholesterol. This pathophysiological mechanism has positioned PCSK9 as a key pharmacological target in the fight against hypercholesterolemia and CVD [63,64,65].

To restore LDLR recycling and promote LDL-C clearance, contemporary therapies target PCSK9 via two principal mechanisms [10,14].

Monoclonal antibodies (e.g., evolocumab, alirocumab)—These agents bind circulating PCSK9, preventing its interaction with LDLR. By blocking this interaction, they preserve receptor recycling and substantially reduce LDL-C levels. However, monoclonal antibodies act only on extracellular PCSK9 and must be administered every 2–4 weeks, posing potential challenges for long-term adherence [10,14,23].

Inclisiran—A fundamentally distinct approach, inclisiran is a chemically stabilized siRNA designed to target PCSK9 mRNA within hepatocytes. Delivered selectively to the liver via GalNAc conjugation, inclisiran triggers RNA interference, promoting degradation of PCSK9 transcripts and thereby suppressing protein synthesis at its source. With reduced intracellular production of PCSK9, fewer LDLRs are misrouted to lysosomes, allowing for greater receptor recycling and enhanced LDL-C clearance. Unlike monoclonal antibodies, inclisiran acts upstream in the PCSK9 pathway, offering a durable effect: a single subcutaneous injection can suppress PCSK9 production for up to six months. This extended duration of action translates into improved treatment adherence and greater convenience for patients [34,59,66].

Therefore, the LDLR occupies a pivotal junction in cholesterol homeostasis (Table 1) [11,14,38,67]:

- When bound to LDL-C, it is recycled and preserved.
- When bound to PCSK9, it is degraded and lost.
- When PCSK9 is silenced—as with inclisiran—the receptor is free to function optimally.

This molecular decision point—between recycling and degradation—has emerged as a potent therapeutic target. By either blocking PCSK9 with monoclonal antibodies or inhibiting its synthesis through RNA interference, clinicians can now modulate plasma cholesterol with unprecedented precision. Ultimately, this is a story of molecular trafficking, competitive binding, and genetic silencing—all converging on a single receptor whose fate determines the fate of circulating LDL-C [11,14,38,64].

### 2.4. Bempedoic Acid

Bempedoic acid is a novel first-in-class oral lipid-lowering agent that selectively inhibits ACL, a key enzyme in the cholesterol biosynthesis pathway [68,69,70]. Its innovative pharmacological profile addresses the limitations of traditional statin therapy, particularly in patients who are statin-intolerant or who require additional LDL-C reduction [71,72]. Due to its liver-selective activation and mechanism of action upstream of the cholesterol biosynthesis pathway, bempedoic acid offers an effective and well-tolerated option for managing hypercholesterolemia, especially in high- and very high-risk cardiovascular populations [73,74,75,76].

#### 2.4.1. Mechanism of Action

Bempedoic acid functions as a prodrug that is selectively activated in the liver by the enzyme very-long-chain acyl-CoA synthetase 1 (ACSVL1), an enzyme that has minimal expression in skeletal muscle [68,77,78]. This liver-specific bioactivation minimizes systemic exposure and significantly reduces the risk of muscle-related adverse effects seen with statins. Once converted to its active form (active metabolite), bempedoyl-CoA inhibits the enzyme ACL, thereby reducing hepatic production of acetyl-CoA, an essential precursor in the synthesis of cholesterol and fatty acids [76]. The decrease in intracellular cholesterol leads to an upregulation of LDLRs, resulting in increased clearance of circulating LDL-C. The upregulation of LDLRs, following the decrease in intracellular cholesterol, is part of a hepatocyte self-regulatory mechanism (negative feedback) with a role in maintaining intracellular lipid homeostasis, including cholesterol. Thus, the liver cells ensure their optimal level of cholesterol for metabolic needs, without producing excessive intracellular cholesterol accumulation [79]. More specifically, the decrease in intracellular cholesterol triggers a gene regulatory cascade mediated by the factor SREBP-2 (Sterol Regulatory Element-Binding Protein 2)—one of the three members of the SREBP family. This is a ubiquitous transcription factor, expressed in most cells, but with particularly intense activity in the liver. SREBP-2 is synthesized, under normal conditions, as an inactive membrane-bound protein attached to the endoplasmic reticulum. In cholesterol-deficient/cholesterol-depleted cells, SREBP-2 is transported to the Golgi apparatus, where it undergoes proteolytic cleavage that releases its active fragment (nSREBP2). Then, nSREBP-2 is translocated to the nucleus, where it binds to the sterol regulatory element (SRE) in the *LDLR* gene promoter, stimulating its transcription [79,80].

Because ACL is positioned upstream of HMG-CoA reductase in the mevalonate pathway, bempedoic acid can be used synergistically with statins or ezetimibe [81]. Additionally, preclinical evidence suggests that bempedoic acid may activate AMP-activated protein kinase (AMPK), potentially contributing to anti-inflammatory and metabolic effects, although the clinical relevance of this mechanism remains under investigation [69,71,72,77,82,83,84].

#### 2.4.2. Therapeutic Indications

Bempedoic acid is indicated for adult patients with primary hypercholesterolemia (both familial and non-familial forms), established ASCVD, or statin intolerance. Its role is increasingly recognized among patients with high and very high cardiovascular risk who do not achieve LDL-C targets with statin treatment alone, providing LDL-C reductions of approximately 17–28% in monotherapy (Appendix A, Table A5) and up to 48% in combination with ezetimibe [75].

In HeFH, in which patients have mutations that affect LDLR function, bempedoic acid can increase LDL-C clearance by upregulating these receptors. In polygenic or non-familial forms of hypercholesterolemia, it represents an effective adjunct when dietary measures and statin therapy are insufficient [72].

For patients with ASCVD, including those with a history of myocardial infarction, stroke, or peripheral arterial disease, bempedoic acid can assist in secondary prevention strategies. This is particularly beneficial for individuals who cannot tolerate high-intensity statin therapy. Clinical trials, such as CLEAR Harmony and CLEAR Outcomes, have shown that bempedoic acid is effective in reducing major cardiovascular events [73].

In individuals with documented statin intolerance, bempedoic acid offers a well-tolerated oral alternative, avoiding skeletal muscle activation due to its liver-specific metabolism. This feature makes it suitable even for patients who cannot tolerate even low doses of statins [85].

In addition, current ESC and ACC/AHA guidelines recommend the use of bempedoic acid for patients who are at very high cardiovascular risk. This includes individuals with diabetes and target organ damage, those with multivessel coronary artery disease, or patients with advanced chronic kidney disease. In these groups, where the target LDL-C levels are often below 55 mg/dL, bempedoic acid presents a practical oral treatment option before considering more aggressive parenteral therapies, such as PCSK9 inhibitors [3,9].

Taken as a whole, the evidence supports that bempedoic acid offers a versatile and affordable treatment option across a wide range of dyslipidemia scenarios, solidifying its place in contemporary cardiovascular risk reduction strategies [3,9,72,73,76,78,86,87].

#### 2.4.3. Safety and Adverse Effects

Bempedoic acid has shown a favorable safety profile, with a low incidence of muscle-related adverse events. Unlike statins, it does not increase the risk of myalgia, myopathy, or rhabdomyolysis, making it a valuable option for patients with a history of statin-associated muscle symptoms [88,89].

Despite its overall favorable safety profile, bempedoic acid is associated with certain adverse effects that require clinical consideration. Notably, it can induce hyperuricemia due to inhibition of renal organic anion transporter 2 (OAT2), which reduces uric acid excretion and increases serum urate levels. This is particularly concerning in patients with a history of gout, as clinical trials have reported a higher incidence of gout flare-ups in those receiving bempedoic acid compared to placebo [78,88]. Another infrequent yet clinically significant adverse event is tendon rupture, observed primarily in older adults or in patients concurrently using corticosteroids or fluoroquinolones. Mild elevations of hepatic transaminases, particularly alanine aminotransferase (ALT) and aspartate aminotransferase (AST), have also been observed, usually asymptomatic and reversible after discontinuation of bempedoic acid treatment. Other commonly reported events, such as nasopharyngitis, urinary tract infections, and back pain, occurred at rates similar to placebo and are generally not considered clinically significant. Overall, bempedoic acid demonstrates a favorable safety profile, particularly when compared to statins, owing to its low incidence of muscle-related toxicity [78,88].

#### 2.4.4. Contraindications

While bempedoic acid is generally well tolerated, several clinical precautions should be considered [89]. Known hypersensitivity to the active substance or any of its excipients constitutes a clear contraindication. Its use is also contraindicated during pregnancy and lactation, consistent with other agents that inhibit endogenous cholesterol synthesis, due to potential risks to fetal lipid metabolism and development. In patients with a history of gout or hyperuricemia, baseline assessment and ongoing monitoring of serum uric acid levels are advised, given the drug’s propensity to elevate urate concentrations. Although not absolutely contraindicated, severe renal impairment (eGFR < 30 mL/min/1.73 m^2^) necessitates close monitoring, as impaired clearance may increase systemic exposure to the drug [73]. Similarly, caution is warranted in patients with advanced hepatic impairment, as clinical data on bempedoic acid use in this population are limited (Appendix A, Table A6). As with all lipid-lowering therapies, regular monitoring of lipid profile, liver enzymes, and uric acid levels is recommended to ensure both efficacy and safety throughout treatment [73,78].

#### 2.4.5. Dosage and Administration

The recommended dose of bempedoic acid is 180 mg orally once daily, with or without food. It is available as monotherapy tablets and as a fixed-dose combination with ezetimibe (10 mg). No dosage adjustment is required based on age, sex, or in the presence of mild-to-moderate renal or hepatic impairment (Appendix A, Table A6 and Table A7) [84].

#### 2.4.6. Drug–Drug Interactions of Bempedoic Acid

Bempedoic acid has a favorable drug–drug interaction profile (Appendix A, Table A3), largely due to its metabolism being independent of the cytochrome P450 system. It is neither a substrate, inducer, nor inhibitor of CYP enzymes, allowing for safer co-administration with a variety of cardiovascular medications [77].

However, bempedoic acid does interact with renal and hepatic transport proteins. It is a weak inhibitor of organic anion transporters OAT2 and OAT3, which may lead to mild increases in serum uric acid and warrants caution in patients with gout or renal dysfunction. Bempedoic acid also inhibits the hepatic uptake transporters OATP1B1 and OATP1B3, which may affect the pharmacokinetics of certain statins. In particular, exposure to simvastatin is increased and therefore its dose should be limited to a maximum of 20 mg/day. Other statins (e.g., pravastatin, atorvastatin, rosuvastatin) may require careful monitoring, especially in elderly patients or those receiving multiple medications [90].

Co-administration with ezetimibe is well tolerated and exhibits a synergistic effect, providing an additional reduction in LDL-C through complementary mechanisms. Bempedoic acid can also be safely combined with PCSK9 inhibitors without requiring dose adjustment. Although interactions with other drug classes (e.g., fibrates, immunosuppressants, nutraceuticals) are theoretically possible, they are generally not considered clinically significant. However, careful evaluation of concomitant therapy is recommended, especially in patients with polypharmacy or complex comorbidities, as well as monitoring of renal function and uric acid when indicated [90].

#### 2.4.7. Pharmacogenetic Considerations

Although bempedoic acid is not currently associated with mandatory pharmacogenetic testing, more and more emerging perspectives highlight the relevance of genetic factors in optimizing its clinical use. The drug acts on ACL, and Mendelian randomization studies have suggested that individuals with genetically reduced expression of ACL may benefit from enhanced LDL-C-lowering effects or pleiotropic effects, including reductions in inflammatory markers such as C-reactive protein (CRP) [91,92,93]. Although ACL ‘s clinical genotyping is not yet a routine practice, the integration of these data in the future could support a more personalized therapeutic approach, especially in patients with metabolic comorbidities [93,94].

Bempedoic acid is activated in the liver by ACSVL1, encoded by the *SLC27A2* gene. This liver-specific activation contributes to its favorable safety profile by limiting exposure to skeletal muscle tissue. Although no clinically significant polymorphisms in *SLC27A2* have been identified to date, interindividual variability in hepatic ACSVL1 expression may influence therapeutic response in rare cases [95].

In FH, particularly among individuals with mutations in *LDLR*, *ApoB*, or *PCSK9*, the response to lipid-lowering therapy can vary significantly. Bempedoic acid, which acts upstream of statins in the cholesterol biosynthesis pathway, may offer clinical benefit in patients with residual LDLR activity. However, its efficacy is likely limited in cases of complete receptor deficiency, such as HoFH. Genetic screening should be considered in individuals with markedly elevated LDL-C, premature ASCVD, or a strong family history, as it may guide personalized therapeutic strategies [95,96].

Bempedoic acid undergoes metabolism primarily via UGT2B7-mediated glucuronidation. While polymorphisms in UGT (UDP-glucuronosyltransferase)-encoding genes are not routinely assessed in clinical practice, they may theoretically affect systemic drug exposure. In addition, bempedoic acid interacts with hepatic and renal transporters, including OATP1B1/1B3 and OAT2/OAT3. Genetic variants in these transport proteins—such as those in *SLCO1B1*—may influence the pharmacokinetics of bempedoic acid and potentially increase the risk of adverse effects, including hyperuricemia [93].

Although not mandatory, certain basic investigations are recommended depending on the patient’s profile: genetic testing for FH in people with elevated LDL-C values or premature CVD, as well as serum uric acid evaluation in patients with gout or kidney disease. Liver and kidney function should be evaluated prior to treatment initiation. In complex or refractory cases, an extensive pharmacogenomic analysis can support individualization of therapy [89].

#### 2.4.8. Special Populations—Pregnancy, Pediatrics, Elderly

The use of bempedoic acid in special populations warrants careful consideration due to limited clinical data outside the adult population with hypercholesterolemia [97,98].

During pregnancy and lactation, bempedoic acid is contraindicated, as inhibition of cholesterol synthesis may interfere with fetal development. Animal studies have demonstrated embryo-fetal toxicity, and in the absence of adequate human data, treatment should be avoided in these settings. Women of childbearing potential should be advised to use effective contraception during therapy and for a period after discontinuation, in accordance with the drug’s elimination profile [97,98].

Bempedoic acid is not approved for use in individuals under 18 years of age. Its safety and efficacy have not been established in the pediatric population, including children and adolescents with FH. Until supportive clinical data become available, the use of bempedoic acid in this age group is not recommended [98].

In older adults, bempedoic acid has demonstrated consistent efficacy and a favorable safety profile. No age-related dose adjustments are necessary. However, due to a slightly increased risk of adverse reactions such as gout and tendon injury, routine monitoring of renal function, uric acid levels, and musculoskeletal symptoms is advised, particularly in patients with polypharmacy or comorbid conditions [97].

Bempedoic acid represents a meaningful addition to the lipid-lowering therapy landscape, particularly for patients who are statin-intolerant or unable to achieve guideline-recommended LDL-C targets with existing treatments. Its liver-specific activation, upstream site of action in the cholesterol biosynthesis pathway, and compatibility with agents such as ezetimibe and PCSK9 inhibitors support its incorporation into modern, risk-based cardiovascular management strategies. With a favorable safety profile and an expanding evidence base, bempedoic acid has emerged as a practical and effective option in the personalized management of dyslipidemia [78].

### 2.5. Lomitapide

Lomitapide is a first-in-class MTP inhibitor and also a small-molecule benzimidazole derivative, originally developed as an orphan drug for the treatment of HoFH. This rare, severe, and life-threatening genetic disorder is characterized by markedly elevated levels of LDL-C from birth and a substantially increased risk of premature ASCVD. In individuals with null mutations in genes essential for LDLR function, such as *LDLR*, *ApoB*, *PCSK9*, or *LDLRAP1* genes, conventional lipid-lowering therapies (e.g., high-intensity statins, ezetimibe, PCSK9 inhibitors) are often inadequate, as they depend on residual LDLR activity [99].

Lomitapide fills a critical therapeutic need in the management of dyslipidemia and HoFH by exerting its lipid-lowering effect independently of the LDLR pathway. It acts by inhibiting MTP, a key enzyme involved in the assembly and secretion of ApoB-containing lipoproteins in both the liver and intestine—namely, very-low-density lipoproteins (VLDL) and chylomicrons [100]. This MTP inhibition results in a profound reduction in the production of VLDL, ultimately leading to significant decreases in plasma levels of LDL-C and other atherogenic lipoproteins. Clinical studies have demonstrated that lomitapide, when used in conjunction with a low-fat diet and standard lipid-lowering treatments, offers a valuable therapeutic option for HoFH patients who are unable to achieve target LDL-C levels through conventional therapies or lipoprotein apheresis alone [101,102,103].

#### 2.5.1. Mechanism of Action

Lomitapide belongs to the class of lipid-lowering agents known as MTP inhibitors, MTP being an intracellular chaperone protein localized primarily in the lumen of the endoplasmic reticulum within hepatocytes and enterocytes. Its physiological role involves the transfer of triglycerides, cholesterol esters, and phospholipids onto ApoB during the assembly of lipoprotein particles [104].

In the liver, MTP is essential for the formation and secretion of VLDL, the primary precursor of LDL. In the small intestine, MTP facilitates the assembly of chylomicrons, which transport dietary triglycerides and cholesterol. By inhibiting MTP, lomitapide blocks the lipidation process of ApoB, thereby preventing the intracellular formation and secretion of ApoB-containing lipoproteins [101]. This blockade leads to a substantial reduction in the plasma concentration of VLDL and, consequently, LDL-C (Appendix A, Table A5). It is important to note that this mechanism acts independently of LDLR function, which differentiates lomitapide from statins and PCSK9 inhibitors, which require at least some residual activity of these receptors to be effective. In this context, lomitapide is particularly advantageous for patients with null or severely defective mutations of the *LDLR* gene [99,101,103].

Furthermore, by reducing the synthesis of ApoB-containing particles, lomitapide may also lower the circulating levels of [Lp(a)] and postprandial lipoproteins, both of which are considered atherogenic. The overall effect is a broad attenuation of lipid burden, contributing to its therapeutic benefit in a high-risk cardiovascular population [103].

#### 2.5.2. Indications and Clinical Efficacy

Lomitapide is approved as a complementary agent to dietary measures and other lipid-lowering therapies in adults with HoFH, with or without LDL apheresis. In clinical studies, lomitapide has demonstrated substantial efficacy, reducing LDL-C levels by 40–60% in a dose-dependent manner (Appendix A, Table A5) [99]. Longitudinal extensions and observational studies from real-world practice confirm the durability of LDL-C reduction in the long term. It is important to note that lomitapide treatment allowed many patients to reduce or even discontinue apheresis sessions and was associated with a lower frequency of cardiovascular events, as well as stabilization of subclinical atherosclerosis. These therapeutic benefits are most pronounced when drug administration is initiated in the early stages of the disease and integrated into a comprehensive lipid-lowering strategy [102,105].

#### 2.5.3. Adverse Effects and Safety Profile

The adverse event profile of lomitapide is closely linked to its mechanism of action, which involves inhibition of lipid absorption and alterations in hepatic lipid metabolism. The most commonly observed side effects are gastrointestinal, including diarrhea, nausea, dyspepsia, flatulence, abdominal bloating, and vomiting. These symptoms are typically more pronounced during the dose-escalation period and tend to correlate with the amount of dietary fat consumed. Therefore, strict adherence to a low-fat diet is essential to minimize gastrointestinal intolerance. With appropriate dietary counseling, these adverse effects often diminish over time, enhancing long-term treatment tolerability [101].

Hepatic safety remains a key consideration in lomitapide therapy. The drug is known to cause elevations in liver enzymes—particularly ALT and AST—which may be transient or sustained, and can occasionally exceed three times the upper limit of normal. Additionally, lomitapide is associated with hepatic steatosis due to intracellular triglyceride accumulation in hepatocytes, a consequence of impaired VLDL secretion. While imaging modalities such as ultrasound and transient elastography often detect increased hepatic fat content, long-term studies have not demonstrated progression to steatohepatitis or liver fibrosis in patients under regular monitoring. To mitigate hepatic risk, liver function tests should be performed prior to initiating lomitapide and repeated at frequent intervals, especially during the dose titration phase (Appendix A, Table A7) [106].

Although uncommon, lomitapide has been linked to cases of more severe hepatotoxicity, including hepatocellular injury that may necessitate temporary or permanent discontinuation of therapy (Appendix A, Table A7). Due to this risk, lomitapide is contraindicated in individuals with moderate-to-severe hepatic impairment, and in patients presenting with persistent, unexplained elevations in liver transaminases. Careful patient selection and ongoing liver function monitoring are essential to ensure safe use of the medication [99,102].

An additional key safety concern with lomitapide therapy is its effect on the absorption of fat-soluble vitamins and essential fatty acids. By inhibiting chylomicron formation, lomitapide reduces intestinal absorption of vitamins A, D, E, and K, as well as omega-3 and omega-6 fatty acids. Without appropriate supplementation and monitoring, this can result in clinically significant deficiencies—manifesting as night blindness (vitamin A), osteomalacia (vitamin D), coagulopathy (vitamin K), and neuromuscular disturbances (vitamin E). To mitigate these risks, patients receiving lomitapide require routine supplementation with vitamin E and essential fatty acids, as well as periodic assessment of fat-soluble vitamin levels to guide further management [107].

Other less common adverse reactions include fatigue, anorexia, weight loss, elevated creatine kinase levels, and alopecia. Mild hematological changes, such as anemia or thrombocytopenia, have been reported rarely during treatment with lomitapide. Overall, adverse events are usually dose-dependent and manageable with careful monitoring and dietary compliance [105].

#### 2.5.4. Contraindications

Lomitapide is contraindicated in several clinical settings due to its mechanism of action and associated safety concerns. Its use is strictly avoided in patients with moderate-to-severe hepatic impairment or in those with persistent, unexplained elevations in liver transaminases, due to its hepatic metabolism and the risk of exacerbating hepatic steatosis. Furthermore, lomitapide is not recommended for individuals with active gastrointestinal disorders—especially those involving malabsorption—as such conditions may amplify the drug’s gastrointestinal adverse effects and further compromise the absorption of essential nutrients, including fat-soluble vitamins and fatty acids [107].

Importantly, lomitapide is contraindicated during pregnancy and lactation due to its critical interference with lipid metabolism, which is essential for fetal growth and development. Preclinical studies have demonstrated teratogenic effects, underscoring the potential risk to the fetus. As a result, women of childbearing potential must use effective contraception throughout the duration of treatment. In addition, lomitapide should not be initiated in individuals with a known hypersensitivity to the active substance or any of its excipients, given the risk of allergic or adverse reactions [107].

#### 2.5.5. Dosage and Administration

Lomitapide therapy should be initiated and supervised by physicians experienced in the treatment of complex lipid disorders. The recommended starting dose is 5 mg once daily, taken on an empty stomach at least two hours after the evening meal to optimize absorption and minimize gastrointestinal side effects. Dose escalation should occur gradually, typically every 2–4 weeks, and be guided by the patient’s LDL-C response, tolerability, and liver enzyme levels. The typical titration sequence involves increasing the dose from 5 mg to 10 mg, 20 mg, 40 mg, and up to a maximum of 60 mg per day, as tolerated. A strict low-fat diet is mandatory throughout treatment to reduce gastrointestinal adverse effects and support efficacy (Table A7). To mitigate the risk of fat-soluble vitamin and essential fatty acid deficiencies, daily supplementation with vitamin E (400 IU) and essential fatty acids is required. Patient education and ongoing nutritional counseling are crucial to ensure adherence and promote long-term safety [107].

#### 2.5.6. Drug Interactions

Lomitapide is extensively metabolized by the cytochrome P450 3A4 (CYP3A4) enzyme, which explains its considerable potential for clinically relevant drug interactions (Appendix A, Table A4). Concomitant administration of strong or moderate CYP3A4 inhibitors such as itraconazole, fluconazole or clarithromycin is strictly contraindicated, as these agents may significantly increase lomitapide plasma concentrations, leading to an increased risk of hepatotoxicity and systemic adverse reactions. Careful medication review and avoidance of CYP3A4 inhibitors are therefore essential components of safe lomitapide use [108].

Lomitapide also acts as an inhibitor of the CYP3A4 enzyme, which is the primary metabolic pathway for several statins. Consequently, caution is warranted when lomitapide is used in combination with statin therapy, particularly simvastatin. Co-administration can lead to elevated plasma concentrations of simvastatin, thereby increasing the risk of statin-associated adverse effects, including myopathy and, in rare cases, rhabdomyolysis. To minimize this risk, the dose of simvastatin should not exceed 20 mg per day when used concomitantly with lomitapide [107,108].

In patients receiving oral anticoagulants such as warfarin, lomitapide may enhance the anticoagulant effect by inhibiting the CYP3A4 enzyme, which contributes to warfarin metabolism. This pharmacokinetic interaction can result in elevated plasma levels of warfarin, thereby increasing the risk of bleeding complications. To ensure therapeutic safety, careful and frequent monitoring of the international normalized ratio is essential when initiating or adjusting lomitapide therapy in patients on warfarin or similar agents [98].

As lomitapide is a sensitive substrate of the CYP3A4 enzyme, concomitant use with CYP3A4 inducers—such as phenobarbital, carbamazepine, rifampicin, or St. John’s wort—can accelerate its hepatic metabolism, leading to reduced plasma concentrations and diminished therapeutic efficacy. To ensure effective lipid control during co-administration, it is advisable to increase the frequency of LDL-C monitoring. Additionally, if the CYP3A4 inducer is discontinued, a reassessment of lomitapide dosing may be required to avoid excessive drug exposure and maintain treatment efficacy [107,109].

Although lomitapide is not itself a substrate of P-gp, it has been shown to inhibit P-gp activity. As a result, concomitant administration may increase the systemic exposure of drugs that are P-gp substrates by enhancing their intestinal absorption. This interaction is particularly relevant for medications such as dabigatran, digoxin, fexofenadine, posaconazole, ranolazine, saxagliptin, and sitagliptin. When used together with lomitapide, careful monitoring for enhanced pharmacologic or adverse effects of these agents may be warranted, and dose adjustments may be necessary depending on the clinical context [110].

Interactions may also occur between lomitapide and oral contraceptives, as co-administration can increase lomitapide plasma concentrations, potentially heightening the risk of adverse effects. To mitigate this interaction, it is recommended to separate the administration of lomitapide and oral contraceptives by at least 12 h. Additionally, alcohol intake should be minimized—or ideally avoided—during lomitapide therapy due to the increased risk of additive hepatotoxicity. Comprehensive medication reconciliation, along with thorough patient education regarding potential interactions and lifestyle modifications, are essential to ensure the safety and effectiveness of lomitapide treatment [98].

#### 2.5.7. Pharmacogenetic Considerations

The genetic basis of HoFH not only shapes the clinical severity of the disease but also plays a critical role in determining therapeutic response. Individuals with null mutations in the *LDLR* gene—resulting in complete loss of LDLR function—typically exhibit poor response to LDLR-dependent therapies such as statins or PCSK9 inhibitors. However, these patients may derive substantial benefit from lomitapide, which lowers LDL-C via an LDLR–independent pathway by inhibiting VLDL assembly and secretion. Conversely, patients with receptor-defective mutations, which preserve partial LDLR activity, may show some degree of response to LDLR-targeted treatments, but lomitapide remains a valuable adjunct for achieving further LDL-C reduction, regardless of underlying genotype [111].

In autosomal recessive hypercholesterolemia, which results from biallelic mutations in the *LDLRAP1* gene impairing the internalization of LDL particles despite structurally intact LDLRs, lomitapide has demonstrated efficacy comparable to that seen in classic HoFH. This effectiveness is attributable to lomitapide’s LDLR–independent mechanism of action. Similarly, rare genetic variants in *ApoB* and *PCSK9* that can phenotypically resemble HoFH are typically associated with variable residual LDLR activity. Despite this heterogeneity, patients with these mutations generally respond well to lomitapide, reinforcing its utility across a spectrum of genetically defined severe hypercholesterolemias [103,111].

Emerging pharmacogenetic data suggest that polymorphisms in genes encoding drug-metabolizing enzymes (e.g., *CYP3A4*) and transporters (e.g., *ABCB1*) may influence individual pharmacokinetics and susceptibility to adverse effects of lomitapide. Although the clinical application of this information remains largely exploratory, future genotyping of such variants may facilitate more personalized dosing strategies and improve treatment safety and efficacy [103].

In the management of familial dyslipidemias, comprehensive genetic profiling plays a pivotal role in confirming the diagnosis, stratifying cardiovascular risk, and guiding individualized treatment strategies. By identifying specific pathogenic variants—such as those in *LDLR*, *ApoB*, *PCSK9*, or *LDLRAP1*—genetic analysis enables a precision medicine approach that supports clinicians in selecting the most appropriate therapies and informs patients about their disease prognosis and therapeutic options. This personalized framework is particularly valuable when considering the use of lomitapide and other lipid-lowering agents, as it allows for well-informed decisions tailored to the genetic and phenotypic characteristics of each patient [107,112].

#### 2.5.8. Special Populations

Lomitapide is contraindicated during pregnancy due to its demonstrated teratogenic potential. Women of childbearing potential must use effective contraception throughout the duration of treatment, and the drug should be discontinued immediately if pregnancy occurs. Its use is also not recommended during breastfeeding, as it is unknown whether lomitapide is excreted into human milk, and potential harm to the neonate cannot be excluded. The safety and efficacy of lomitapide have not been established in pediatric populations, and its regulatory approval is currently restricted to adults aged 18 years and older. Any off-label use in children should be limited to highly specialized settings and undertaken with expert supervision. In elderly patients (≥65 years), available clinical data do not suggest significant differences in efficacy or safety compared to younger adults. However, due to the increased likelihood of hepatic impairment, comorbid conditions, and polypharmacy in this age group, careful monitoring of liver function and drug interactions is recommended during dose escalation (Appendix A, Table A6 and Table A7) [98,113].

Lomitapide has become an important therapeutic option in the treatment of HoFH, offering substantial reductions in LDL-C levels in patients who often respond poorly to conventional lipid-lowering therapies. Its LDLR–independent mechanism of action makes it particularly valuable in individuals with LDLR-negative genotypes, where other treatments may be ineffective. Although hepatic and gastrointestinal adverse effects warrant careful monitoring, long-term data support the sustained efficacy and manageable safety profile of lomitapide when used appropriately.

As advances in genetics continue to refine the diagnosis and classification of FH and related dyslipidemias, lomitapide exemplifies the potential of precision pharmacotherapy. Integrating pharmacogenetic insights with clinical parameters into individualized treatment strategies will further enhance outcomes and safety in patients with severe inherited lipid disorders [103,111].

### 2.6. Angiopoietin-like Protein 3 Inhibitors

ANGPTL3 inhibitors represent a novel class of lipid-lowering agents that target lipid metabolism through a mechanism distinct from traditional therapies such as statins or PCSK9 inhibitors. Angiopoietin-like proteins (ANGPTL) comprise a group of proteins that involve members 1–8 of angiopoietins, with distinctions regarding tissue regulation and expression. Each of them includes a shared domain at the amino terminus, a fibrinogen-like domain at the C-terminus of the carboxyl, a binding region, and a helical domain [114,115]. ANGPTL3 is a liver-secreted protein that plays a critical role in the regulation of plasma lipids by inhibiting lipoprotein lipase (LPL) and endothelial lipase (EL), key enzymes involved in the catabolism of triglyceride-rich lipoproteins and HDL metabolism, respectively. Genetic studies have shown that individuals with loss-of-function mutations in the *ANGPTL3* gene exhibit significantly lower levels of LDL-C, triglycerides, and HDL-C, and a reduced risk of ASCVD, highlighting ANGPTL3 as a promising therapeutic target [116,117,118,119,120].

#### 2.6.1. Mechanism of Action

ANGPTL3 inhibitors function by neutralizing ANGPTL3 activity, thereby enhancing the activity of LPL and EL. ANGPTLs form a distinct family of proteins with multiple roles in biological and pathological processes, encompassing hormone regulation, glucose metabolism, and insulin sensitivity [121]. LPL acts as a part of the lipase class found on the luminal surface of capillaries which catabolizes plasma triglycerides from lipoproteins including chylomicrons and VLDL. Low levels of LPL cause severe hypertriglyceridemia and decreased HDL levels, thereby elevating the risk of ischemic heart disease (Appendix A, Table A5). ANGPTLs represent a group of proteins capable of mediating post-translational regulation of LPL, inhibiting it [122]. Therefore, the pharmacologic inhibition of ANGPTL3 will lead to the following: a. enhanced LPL and EL activity; b. reduction in VLDL, LDL-C, triglycerides and ApoB-containing particles; c. increased clearance of remnant lipoproteins [123]. This mechanism works independently of LDLR, which is a key therapeutic advantage.

The monoclonal antibody evinacumab is currently the most advanced ANGPTL3-targeted therapy, approved by the FDA for use in patients with HoFH. Unlike statins or PCSK9 inhibitors, as already mentioned, ANGPTL3 inhibition reduces LDL-C levels independently of LDLR activity, making it particularly effective in patients with complete or near-complete loss of LDLR function, such as those with HoFH [123,124].

#### 2.6.2. Therapeutic Indications

Evinacumab is indicated as an adjunct to other lipid-lowering therapies for patients with HoFH who are unable to achieve adequate LDL-C reduction with conventional treatments. Clinical trials have demonstrated LDL-C reductions of approximately 47% in this population, along with significant decreases in triglycerides and non-HDL-C [123]. The unique LDLR-independent mechanism allows evinacumab to be effective where other treatments have limited efficacy. While current approval is limited to HoFH, ongoing studies are investigating its role in broader dyslipidemia populations, including severe/refractory hypertriglyceridemia and mixed dyslipidemia. More specifically, patients who do not reach LDL-C target with statins, ezetimibe or PCSK9 inhibitors may benefit form additional LDL-lowering through ANGPTL3 inhibition as they can reduce LDL-C, triglycerides and non-HDL cholesterol without increasing myopathy risk. The 2019 ESC/EAS Guidelines for the management of dyslipidaemias recognize the need for emerging therapies in patients with HoFH and high residual risk despite optimal statin and ezetimibe therapy. ANGPTL3 inhibitors are noted as a promising approach in this high-risk group [124,125]. Similarly, the 2022 ACC Expert Consensus Decision Pathway on the Role of Nonstatin Therapies in LDL-C Lowering identifies evinacumab as a potential adjunct in patients with HoFH or those with genetically confirmed lipid disorders who fail to achieve target LDL-C levels [126].

Several investigations have contributed to the evidence that evinacumab can successfully achieve baseline LDL optimization in patients diagnosed with HoFH and HeFH, without LDLR mutations [34,127]. This therapeutic mechanism provides a targeted approach to the treatment of individuals with LDLR deficient who demonstrate resistance against other antilipid drugs, such as PCSK9 and HMG-CoA reductase inhibitors. Recent approval has been attributed to the ANGPTL3 inhibitor being prescribed in addition to aggressive lipid-lowering therapy in pediatric patients diagnosed with HoFHaged aged 5 years old in the United States (US) and 6 months or older in (European Union) EU [128,129,130].

Moreover, real-world data and post-marketing experience are expected to further elucidate the benefit of ANGPTL3 inhibition in high-risk patients, including those with residual cardiovascular risk despite optimal statin therapy. Given its impact on multiple lipid fractions, evinacumab may also have a role in conditions such as familial combined hyperlipidemia and refractory hypertriglyceridemia, although these applications remain under investigation [131,132,133].

#### 2.6.3. Safety and Adverse Effects

ANGPTL3 inhibitors such as evinacumab have demonstrated a favorable safety profile in clinical trials. The most commonly reported adverse events include nasopharyngitis, influenza-like symptoms, dizziness, and infusion-related reactions. These events are typically mild to moderate in severity [123,134]. There have been no significant reports of hepatotoxicity or muscle-related side effect, distinguishing ANGPTL3 inhibitors from statins (Appendix A, Table A7). However, as a monoclonal antibody requiring intravenous administration, evinacumab necessitates monitoring for hypersensitivity and infusion-related complications, especially during initial infusions [135].

Longer-term safety monitoring is still needed to assess the risk of immune reactions, potential changes in lipid-soluble vitamin absorption, and effects on lipid metabolism in special populations. Currently available data do not suggest any significant increase in the risk of new-onset diabetes or hepatic enzyme elevations [136,137].

#### 2.6.4. Contraindications

Evinacumab is contraindicated in individuals with known hypersensitivity to the active substance or any of its excipients. Caution is advised in patients with a history of allergic reactions to monoclonal antibodies. Data on use in pregnancy and lactation are limited, and given the potential effects on lipid metabolism during fetal development, use should be avoided unless clearly necessary (Appendix A, Table A6). Safety data of evinacumab is currently available for children above 1-year-old, pending further safety data [128,130].

#### 2.6.5. Dosage and Administration

The recommended dose of evinacumab for patients with HoFH is 15 mg/kg administered as an intravenous infusion over 60 min once every four weeks. No dosage adjustment is required based on age, sex, or in the presence of mild-to-moderate renal or hepatic impairment (Appendix A, Table A6 and Table A7). Due to limited data in patients with severe hepatic impairment, caution is advised, and clinical monitoring is recommended [128,130].

Patient adherence may benefit from the relatively infrequent dosing schedule, which may be an advantage over agents requiring more frequent administration. Nevertheless, logistical challenges associated with intravenous administration should be considered in long-term management planning [138].

#### 2.6.6. Drug–Drug Interactions

Evinacumab does not undergo metabolism via cytochrome P450 enzymes and does not interfere with common hepatic transporters, thus minimizing the potential for drug–drug interactions. Thus, evinacumab can be safely co-administered with statins, ezetimibe, PCSK9 inhibitors, and other lipid-modifying agents. However, data are limited on interactions with immunosuppressants, antidiabetic medications, or other monoclonal antibodies, and careful assessment is warranted in complex polypharmacy scenarios [139,140,141].

#### 2.6.7. Pharmacogenetic Considerations

Current use of ANGPTL3 inhibitors does not involve pharmacogenetic testing. However, genetic screening may aid in identifying suitable candidates, particularly individuals with HoFH or unexplained combined dyslipidemia. Loss-of-function mutations in *ANGPTL3* gene have been linked to lifelong reductions in atherogenic lipoproteins and cardiovascular risk, reinforcing the therapeutic rationale behind ANGPTL3 inhibition [142].

Additionally, *ANGPTL3* genetic variants may interact with other lipid-related genes such as *APOC3*, *LPL*, and *APOA5*, influencing the individual lipid phenotype and response to therapy. Further pharmacogenomic research may support the development of tailored approaches in complex dyslipidemia cases [143,144].

#### 2.6.8. Special Populations

The safety and efficacy of evinacumab in pregnancy, lactation, and pediatric populations under 1-year-old remain unknown. Use in older adults has not demonstrated any significant differences in tolerability or efficacy, and no dose adjustments are necessary. In patients with severe renal or hepatic impairment, clinical discretion is advised due to limited data (Appendix A, Table A6) [145].

For patients with coexisting metabolic syndrome or diabetes mellitus, the impact of ANGPTL3 inhibition on insulin sensitivity and glucose metabolism appears neutral, with no current evidence of adverse glycemic effects. However, ongoing studies are evaluating potential metabolic benefits in this subgroup [137,146,147,148].

ANGPTL3 inhibitors, particularly evinacumab, offer a novel and effective treatment strategy for patients with severe or refractory dyslipidemia, especially those with HoFH. Their LDLR-independent mechanism, favorable safety profile, and potential for use alongside existing lipid-lowering therapies position them as a valuable tool in personalized cardiovascular risk management. As clinical experience and data expand, ANGPTL3 inhibition may play an increasingly important role in the therapeutic landscape of lipid disorders, particularly for patients with limited therapeutic options or complex lipid phenotypes [114,149,150].

### 2.7. Pharmacovigilance Aspects

Post-marketing and long-term clinical studies have reinforced the safety and efficacy profiles of modern lipid-lowering therapies. Statins remain the cornerstone, supported by outcome trials such as HPS and JUPITER, which confirmed significant reductions in cardiovascular morbidity and mortality. Ezetimibe has demonstrated incremental benefit in secondary prevention, particularly in the IMPROVE-IT trial [151]. PCSK9 monoclonal antibodies (evolocumab and alirocumab) have shown durable LDL-C reductions and cardiovascular benefit in FOURIER and ODYSSEY OUTCOMES, with long-term extensions such as FOURIER-OLE and ODYSSEY APPRISE confirming sustained efficacy and safety [24,25,152,153,154]. Inclisiran, though newer, has robust phase III data (ORION-9, -10, -11) and ongoing cardiovascular outcome evaluation in the ORION-4 trial [155,156]. Lomitapide has been followed in the LOWER registry, providing real-world insights into long-term use in HoFH [157]. Evinacumab, approved for HoFH, has demonstrated promising results in the EVOPACS trial and in long-term extension studies of the ELIPSE program [123,158].

## 3. Overview of the Major Guidelines Recommendations on Lipid-Lowering Therapies

The initiation and escalation of lipid-lowering therapies are guided by comprehensive recommendations from international societies, which emphasize a stratified approach according to cardiovascular risk categories and specific LDL-C thresholds (Table 2). The 2019 ESC/EAS Guidelines advocate a stepwise intensification strategy in which high-intensity statins are first-line therapy across all risk levels. For patients classified as very high risk, such as those with established atherosclerotic cardiovascular disease (ASCVD), diabetes mellitus with target organ damage, or severe chronic kidney disease, an LDL-C goal of <55 mg/dL (1.4 mmol/L) and a reduction of at least 50% from baseline are recommended. If this target is not achieved with maximally tolerated statin therapy, ezetimibe is added as the next step, followed by the introduction of PCSK9 inhibitors when necessary to reach treatment goals [9].

The 2018 ACC/AHA Guidelines similarly endorse high-intensity statin therapy as the cornerstone of management but define slightly less stringent LDL-C targets in very high-risk patients, recommending an LDL-C level <70 mg/dL. These guidelines also encourage clinician-patient discussion before initiating PCSK9 inhibitors, emphasizing considerations of cost and patient preference [159].

In contrast, the National Institute for Health and Care Excellence (NICE) Guidelines adopt a more conservative approach, prioritizing high-intensity statins and reserving ezetimibe and PCSK9 inhibitors primarily for patients who do not achieve at least a 40% reduction in non-HDL-C or fail to reach individualized LDL-C targets despite optimized statin therapy. NICE also incorporates formal health-economic evaluations into treatment algorithms, which results in more restrictive eligibility criteria for PCSK9 inhibitor reimbursement compared to ESC/EAS or ACC/AHA recommendations [8].

These guideline frameworks collectively reflect a convergence toward intensive lipid lowering in high-risk populations while underscoring differences in treatment thresholds, reimbursement policies, and resource allocation across healthcare systems.

## 4. Future Perspectives in Cholesterol-Lowering Therapy

Despite significant advances in the management of dyslipidemia, a substantial proportion of patients fail to reach guideline-recommended LDL-C targets or are intolerant to statin therapy. Future lipid-lowering strategies aim to improve efficacy, tolerability, and adherence through the development of novel agents that support an individualized therapeutic approach. Among the most promising innovations are oral PCSK9 inhibitors, such as MK-0616 (Merck) and AZD0780 (AstraZeneca), which have demonstrated LDL-C reductions of up to 61% in early-phase clinical trials and offer the convenience of oral administration compared to injectable monoclonal antibodies or siRNA-based therapies. Gene-editing approaches, including base editing platforms such as VERVE-101 and VERVE-102, enable permanent inactivation of the *PCSK9* gene in hepatocytes, with single-dose infusions achieving sustained LDL-C reductions (~55–60%) and favorable early safety profiles, particularly relevant for patients with familial hypercholesterolemia. In parallel, oral fixed-dose combinations, such as bempedoic acid plus ezetimibe (marketed as Nexlizet in the U.S.), provide synergistic LDL-C-lowering (~48%) and anti-inflammatory effects (30–35% hsCRP reduction), especially suitable for statin-intolerant patients. CETP (Cholesteryl Ester Transfer Protein) inhibitors, once considered a failed class, have re-emerged with improved safety and efficacy; obicetrapib has shown ~45–50% LDL-C reduction and is currently under evaluation in cardiovascular outcome trials. Additionally, RNA-based ANGPTL3 inhibitors, such as solbinsiran, are in early clinical stages and may offer long-term therapeutic options for patients with severe or genetic forms of hypercholesterolemia, particularly those unresponsive to PCSK9 inhibition. Finally, PCSK9 vaccination—a preclinical approach using virus-like particles (VLPs)—has shown promise in inducing long-lasting LDL-C reductions with minimal dosing frequency, potentially transforming long-term lipid control. Collectively, these next-generation therapies reflect a shift toward more durable, precise, and patient-tailored lipid-lowering strategies, with the potential to significantly improve cardiovascular outcomes in high-risk populations [160,161].

## 5. Conclusions

The management of hypercholesterolemia has entered a transformative era, propelled by deeper insights into lipid metabolism and the advent of innovative therapies that extend well beyond the scope of conventional statins. This review underscores the expanding therapeutic arsenal—including PCSK9 inhibitors, siRNA-based agents such as inclisiran, ACL inhibitors like bempedoic acid, MTP inhibitors, and ANGPTL3 inhibitors—each offering unique mechanisms of action, distinct efficacy profiles, and tailored safety considerations.

Collectively, these emerging strategies not only provide valuable alternatives for patients with FH or statin intolerance but also represent promising tools to mitigate persistent residual cardiovascular risk in complex, high-risk populations. Their integration into individualized treatment algorithms—guided by pharmacogenomics, refined risk stratification, and patient preferences—signals a paradigm shift toward more precise and personalized lipid management.

As the field continues to evolve, sustained research efforts, robust real-world evidence, and thoughtful evaluations of long-term outcomes and cost-effectiveness will be essential to define the optimal role of these agents within contemporary and future dyslipidemia care pathways.

## Figures and Tables

**Table 1:** LDL-receptor “fate”: a comparison of LDL-C, PCSK9, and inclisiran [11,14,38,64].

Feature	LDL-C	PCSK9	Inclisiran
Molecule type	Lipoprotein particle (contains ApoB-100)	Secreted protein regulator (serine protease)	Chemically modified small interfering RNA (siRNA)
Binds to LDLR	Yes, via LA repeats (extracellular)	Yes, via EGF-A domain (extracellular or intracellular)	No direct binding; acts upstream at the mRNA level
Binding site on LDLR	LA (ligand-binding) domain (R4–R7)	EGF-A domain	None
Ca^2+^ dependence	Required for structural stability of LA repeats	Ca^2+^ required to fold EGF-A, but not in binding interface	Not applicable
Effect of endosomal pH	Acidic pH weakens interaction → dissociation	Acidic pH strengthens interaction → complex stabilized	Prevents PCSK9 protein formation → no interaction occurs
LDLR fate	Recycled to membrane	Degraded in lysosome	Preserved (prevents degradation indirectly)
LDL-C clearance	Enhanced	Inhibited	Enhanced
Mechanism of action	Delivers cholesterol to liver cells for degradation	Tags LDLR for lysosomal degradation	Silences hepatic *PCSK9* gene expression (RNA interference)
Therapeutic role	Target of LDLR function (not a therapy)	Targeted by inhibitors (e.g., monoclonal antibodies)	Therapeutic agent (e.g., inclisiran)
Route of action	Natural physiological ligand	Endogenous negative regulator	Synthetic therapeutic siRNA
Time of action	Fast (minutes)	Moderate (hours)	Long-term (weeks to months per dose)
Clinical outcome	Lowers plasma LDL-C when cleared by LDLR	Raises plasma LDL-C by reducing LDLR availability	Lowers LDL-C by increasing LDLR availability
Example drugs	Not applicable	Evolocumab, alirocumab	Inclisiran

Note: LDL-C is the native ligand, whose removal depends on LDLR availability and recycling. PCSK9 is the endogenous inhibitor, promoting LDLR degradation and limiting cholesterol clearance. Inclisiran is the genetic silencer, preventing PCSK9 production altogether and indirectly boosting LDLR recycling and function.

**Table 2:** Comparative overview of major guidelines (ESC/EAS, ACC/AHA, NICE) on lipid-lowering therapies including emerging agents [8,124,159].

Therapy Class	ESC/EAS	ACC/AHA	NICE Guidelines
Statins	First-line therapy across all risk categories. High-intensity statins recommended for high and very-high-risk patients.	First-line therapy. High-intensity statins preferred. No fixed LDL-C target; emphasis on ≥50% LDL-C reduction.	First-line therapy. High-intensity statins recommended. Target: ≥40% non-HDL-C reduction.
Ezetimibe	Second-line if LDL-C target not achieved on statins. Combination strongly recommended.	Added if LDL-C remains ≥70 mg/dL in very high-risk patients on maximal statins.	Added if target LDL-C reduction not achieved. Approved for combination or monotherapy if statin intolerant.
PCSK9 Inhibitors	Third-line after statin + ezetimibe in very-high-risk patients. Recommended if LDL-C ≥55 mg/dL persists.	Reasonable if LDL-C ≥70 mg/dL on maximal statin + ezetimibe in very high-risk individuals.	Approved for FH or very-high-risk patients who do not achieve ≥40% non-HDL-C reduction. Strict eligibility and cost control.
Inclisiran	Recognized as an alternative to PCSK9 inhibitors in high/very-high-risk patients; recommended in updated position statements.	Mentioned as promising but limited outcome data; not incorporated into formal treatment pathways.	Approved for primary hypercholesterolemia or mixed dyslipidemia in adults if LDL-C goals unmet on maximally tolerated therapy.
Bempedoic Acid	Considered as adjunctive therapy in patients with statin intolerance or inadequate LDL-C reduction; acknowledged in 2023 updates.	Emerging option for statin-intolerant patients or those requiring further LDL-C lowering; outcome data evolving.	Approved for use in statin-intolerant patients or as add-on therapy. Cost-effectiveness considerations apply.
ANGPTL3 Inhibitors (Evinacumab)	Approved in HoFH with inadequate LDL-C response to other treatments; reserved for specialist care in severe cases.	Recognized as adjunctive treatment in HoFH; availability varies.	Not routinely commissioned; considered only in HoFH under specialist supervision and exceptional circumstances.
Lomitapide	Approved in HoFH; recommended under expert supervision due to hepatic fat accumulation risk and monitoring requirements.	Approved for HoFH; reserved for specialized lipid clinics.	Commissioned only for HoFH in highly selected patients; requires specialist management.

## Data Availability

No new data were created or analyzed in this study. Data sharing is not applicable.

## Abbreviations

The following abbreviations are used in this manuscript:

ACC	American College of Cardiology
ACL	Adenosine triphosphate-citrate lyase
ACSVL1	Very-long-chain acyl-CoA synthetase 1
AHA	American Heart Association
ALT	Alanine aminotransferase
AMPK	AMP-activated protein kinase
ANGPTL3	Angiopoietin-like protein 3
ANP	Atrial Natriuretic Peptide
ApoB	Apolipoprotein B
ASCVD	Atherosclerotic cardiovascular disease
ASGPR	Asialoglycoprotein receptor
AST	Aspartate aminotransferas
BCRP	Breast Cancer Resistance Protein
CETP	Cholesteryl Ester Transfer Protein
CRP	C-reactive protein
CVD	Cardiovascular diseases
CYP3A4	Cytochrome P450 3A4
DNA	Deoxyribonucleic acid
eGFR	Estimated glomerular filtration rate
EAS	European Atherosclerosis Society
EL	Endothelial lipase
EGF-A	Epidermal growth factor-like repeat A
ESC	European Society of Cardiology
EU	European Union
FDA	Food and Drug Administration
FH	Familial hypercholesterolemia
HDL	High-density lipoprotein
GalNAc	N-acetylgalactosamine
hs-CRP	High-sensitivity C-reactive protein
HDL-C	High-density lipoprotein cholesterol
HeFH	Heterozygous familial hypercholesterolemia
HMG-CoA	3-hydroxy-3-methylglutaryl coenzyme A
HoFH	Homozygous familial hypercholesterolemia
INR	International Normalized Ratio
LA	Ligand-binding type A
LDL	Low-density lipoprotein
LDL-C	Low-density lipoprotein cholesterol
LDLR	Low-density lipoprotein receptor
Lp(a)	Lipoprotein(a)
LPL	Lipoprotein lipase
mRNA	messenger ribonucleic acid
MTP	Microsomal triglyceride transfer protein
NICE	National Institute for Health and Care Excellence
OAT	Organic anion transporter
PCSK9	Proprotein convertase subtilisin/kexin type 9
P-gp	P-glycoprotein
RISC	RNA-induced silencing complex
RNA	Ribonucleic acid
RNAi	RNA interference
siRNAs	Small interfering RNAs
SRE	Sterol regulatory element
SREBP	Sterol Regulatory Element-Binding Protein
TGs	Triglycerides
UGT	UDP-glucuronosyltransferase
US	United States
VEGF	Vascular endothelial growth factor
VLDLs	Very-low-density lipoproteins
VLP	Virus-like particle

## Appendix A

**Table A1:** PCSK9 inhibitor drug–drug interactions [17,18].

Interacting Drug/Class	Type of Interaction	Effect	Clinical Recommendation
Statins	Pharmacodynamic (additive effect)	Additive LDL-C lowering effect	Commonly co-administered; no adjustment needed
Ezetimibe	Pharmacodynamic (additive effect)	Enhanced LDL-C reduction	Safe and often used in combination
Immunosuppressants (e.g., cyclosporine)	Theoretical (immune modulation)	Potential modulation of antibody metabolism (no known interaction)	Monitor if used concurrently, though no evidence of harm
Anticoagulants/Antiplatelets	Theoretical (safety concern)	Very rare bleeding events reported in trials (no causal link)	Use with vigilance in high-risk patients
Vaccines/Biologics	Theoretical (immune system impact)	Limited data; no immunosuppression by PCSK9 inhibitors	No adjustments necessary; standard vaccination practices apply
Other monoclonal antibodies	Theoretical (anti-drug antibody risk)	No known interactions	Monitor for unexpected immune responses if used concurrently
Pregnancy/lactation drugs	None known (monoclonal IgG1-based)	Large molecules; minimal placental transfer early in pregnancy	Use only if clearly needed; no DDI concerns

Note: PCSK9 inhibitors are not metabolized by CYP450 enzymes, nor do they affect transporter proteins like OATP or P-gp, so they are not expected to interact with most oral drugs.

**Table A2:** Inclisiran drug–drug interactions [33,42,49,50].

Interacting Drug/Class	Type of Interaction	Effect	Clinical Recommendation
Statins	Pharmacodynamic (additive effect)	Additive LDL-C lowering effect	Co-administration is common and safe
Ezetimibe	Pharmacodynamic (additive effect)	Further LDL-C reduction	Safe; often used in combination
Antiplatelet/anticoagulants	Theoretical (injection site bleeding)	Slight ↑ risk of local bleeding due to subcutaneous administration	Monitor injection site; otherwise safe
CYP450 substrates/inhibitors	No interaction	Inclisiran is not metabolized by or affecting CYP enzymes	No adjustment needed
Renally excreted drugs	No interaction	Although inclisiran is partially cleared renally, no accumulation observed	No adjustment needed in mild/moderate renal impairment
Other siRNA or RNA-based therapies	Theoretical (immune stimulation)	Potential for unknown immune or off-target RNA effects	Monitor when used concurrently, though data are limited
Immunosuppressants (e.g., cyclosporine)	Theoretical (PK/PD interactions)	No known interaction; limited clinical data	Caution advised; monitor if used together

Note: Inclisiran has minimal interaction potential due to no involvement with CYP450 or transporter systems (e.g., OATP, P-gp). Subcutaneous route and hepatocyte-specific uptake via ASGPRs. Long dosing interval (every 6 months). Legend: ↑—increase.

**Table A3:** Bempedoic acid drug–drug interactions [77,90].

Interacting Drug	Type of Interaction	Effect	Clinical Recommendation
Simvastatin	Pharmacokinetic (increased exposure)	↑ Simvastatin levels (dose-dependent) → risk of myopathy/rhabdomyolysis	Avoid doses >20 mg/day when co-administered
Pravastatin	Pharmacokinetic (increased exposure)	↑ Pravastatin levels (dose-dependent) → risk of myopathy	Avoid doses >40 mg/day
Atorvastatin/Rosuvastatin	Minimal interaction	No clinically meaningful interaction	No clinically relevant interaction expected, although monitoring is advisable in elderly or polytreated patients
Colchicine	Additive toxicity (e.g., myopathy)	Potential ↑ risk of myopathy when used with statins and bempedoic acid	Monitor closely for muscle symptoms
Fibrates (e.g., gemfibrozil)	Pharmacodynamic/toxicity overlap	↑ Risk of myopathy when combined with statins and bempedoic acid	Caution advised; monitor for muscle-related side effects
Uricosuric agents (e.g., probenecid)	Pharmacodynamic	Opposing effects on uric acid (bempedoic acid may ↑ uric acid)	Monitor serum uric acid levels
Cyclosporine	Unclear mechanism	Potential ↑ bempedoic acid exposure	Use caution; consider monitoring bempedoic acid-related effects

Note: Bempedoic acid inhibits OATP1B1 and OATP1B3, contributing to interactions with statins that are OATP substrates. Legend: ↑—increase.

**Table A4:** Lomitapide drug–drug interactions [98,108].

Interacting Drug/Class	Type of Interaction	Effect	Clinical Recommendations
Strong CYP3A4 inhibitors (e.g., ketoconazole, clarithromycin, itraconazole)	CYP3A4 inhibition	↑ Lomitapide plasma concentration → risk of hepatotoxicity	Contraindicated
Moderate CYP3A4 inhibitors (e.g., fluconazole, diltiazem, verapamil)	CYP3A4 inhibition	↑ Lomitapide exposure	Reduce lomitapide dose; monitor liver enzymes
Weak CYP3A4 inhibitors (e.g., atorvastatin)	CYP3A4 inhibition	Mild ↑ in lomitapide levels	May require dose adjustment or close monitoring
CYP3A4 inducers (e.g., rifampin, carbamazepine, St. John’s Wort)	CYP3A4 induction	↓ Lomitapide levels → reduced efficacy	Avoid if possible
Statins (especially simvastatin, lovastatin)	Additive hepatotoxicity risk	↑ Risk of transaminase elevations	Use lowest effective statin dose; monitor LFTs closely
Warfarin	Lomitapide inhibits CYP2C9	↑ INR and bleeding risk	Monitor INR more frequently after starting or adjusting lomitapide
Oral contraceptives (estrogen-containing)	Hepatotoxicity risk overlap	↑ Risk of liver enzyme elevation	Monitor LFTs; consider non-estrogen contraceptive
Vitamin E and fat-soluble vitamins	Fat malabsorption due to lomitapide	Deficiency risk (esp. A, D, E, K)	Recommend daily multivitamin supplement with fat-soluble vitamins
P-gp substrates (e.g., digoxin, colchicine)	Lomitapide inhibits P-gp	↑ Substrate levels → potential toxicity	Use caution; monitor drug levels or adverse effects
Alcohol	Additive hepatic toxicity	↑ Risk of hepatotoxicity	Advise limiting or avoiding alcohol

Note: Lomitapide is a strong inhibitor of CYP3A4 and P-gp substrate. Due to its boxed warning for hepatotoxicity, many interactions are driven by increased liver injury risk. Liver function tests (ALT, AST, bilirubin) must be monitored frequently, especially with interacting drugs. Fat-soluble vitamin deficiency is common; supplementation is essential. Legend: ↑—increase; ↓—decrease.

**Table A5:** Comparative effects of most used LDL-lowering agents on major lipid parameters [14,73,78,83,96,99,102,151,152,156].

Drug/Class	LDL-C	HDL-C	TG	Other Clinically Relevant Effect *
Statins (moderate–high intensity)	30–60% [99]	5–10% [99]	10–40% (greater fall when baseline TG is high) [99]	↓ hs-CRP ≈ 30–40% [99]
Ezetimibe	18–25% [99]	2–3% [151]	5–10% [151]	↓ hs-CRP ≈ 8–10% [151]
PCSK9 mAbs (alirocumab/evolocumab)	50–60% (on top of statin) [152]	10–15% [152]	15–30% [152]	↓ Lp(a) ≈ 20–30% [14]
Inclisiran (siRNA)	45–52% [156]	5–8% [156]	10–30% (real-world data) [156]	↓ Lp(a) ≈ 20–25% [156]
Bempedoic acid	17–23% [73]	≈5% (small fall) [78]	≈0% (neutral) [78]	↓ hs-CRP ≈ 25–30% [83]
Lomitapide	40–60% (HoFH trials up to −70%) [102]	~0% (no consistent change) [102]	30–65% (dose-dependent) [102]	↓ Apo B ≈ 50–56% [96]

Note 1: * “Other” lists the best-documented additional biomarker most likely to influence cardiovascular risk for that agent. Note 2: Values are average placebo-corrected changes seen in pivotal phase III trials (or large real-world cohorts where noted). Ranges reflect different doses, backgrounds (e.g., with/without statin), and patient phenotypes. All agents lower non-HDL-C proportionally to LDL-C; ApoB reductions generally mirror LDL-C reductions except where specified. Note 3: Potency hierarchy for LDL-C: PCSK9 mAbs ≈ inclisiran > high-intensity statins > lomitapide (in HoFH) > bempedoic acid ≈ ezetimibe. Inflammation marker (hs-CRP) is uniquely lowered by statins and bempedoic acid; PCSK9-pathway drugs have minimal direct effect. Lp(a) lowering is meaningful only with PCSK9 inhibition (antibody or siRNA). Triglyceride impact is modest for most except lomitapide (designed to block VLDL production). Legend: ↓—decrease.

**Table A6:** Dose adjustments of hypolipidemic drugs in renal impairment [18,32,34,52,88,96,112,151].

Drug/Class	Renal Elimination	Dose Adjustment in Mild/Moderate Impairment (eGFR 30–89)	Dose Adjustment in Severe Impairment (eGFR < 30)	Observations
Statins	Variable (some renal excretion)	Usually no adjustment for atorvastatin, fluvastatin, pitavastatin. Yes for others.	Yes for rosuvastatin, simvastatin, pravastatin—reduce dose.	Choose statin based on renal profile; monitor CK and renal function.
Ezetimibe	Minimal (mainly fecal)	No adjustment needed.	No adjustment needed.	Safe in all stages of CKD; may be used in dialysis patients.
PCSK9 Inhibitors (alirocumab, evolocumab)	Minimal renal clearance	No adjustment needed.	No adjustment needed.	No clinically significant change in exposure. Safe in severe CKD.
Inclisiran	Partial renal clearance (~20%)	No adjustment in mild/moderate impairment.	Use with caution; no dosage adjustment recommended but data limited.	Exposure may increase slightly in severe CKD; monitor as needed.
Bempedoic Acid	Renal + hepatic elimination	No adjustment in mild/moderate impairment.	Caution advised; no adjustment officially required, but monitor.	Limited data in eGFR <30; risk of ↑ uric acid.
Lomitapide	Minimal renal excretion	No adjustment recommended.	Avoid in severe renal impairment due to lack of data.	Contraindicated or not recommended in severe CKD; monitor LFTs and lipid response.

Note: Atorvastatin is preferred in renal impairment due to minimal renal clearance. Rosuvastatin requires dose reduction in eGFR < 30 (max 10 mg/day). Inclisiran and bempedoic acid do not require dose changes, but monitoring is advised in eGFR <30 due to limited trial data. Lomitapide has no official renal clearance concerns but is not recommended in severe CKD due to safety uncertainties. Legend: ↑—increase.

**Table A7:** Dose adjustments of hypolipidemic drugs in liver impairment [18,32,34,52,88,96,112,151].

Medicine/Class	Hepatic Metabolism	Mild Impairment (Child-Pugh A)	Moderate Impairment (Child-Pugh B)	Severe Impairment (Child-Pugh C)	Observations
Statins (varies by type)	Extensive (CYP3A4/2C9) or glucuronidation	Use with caution, start low	Avoid or reduce dose, especially for simvastatin, lovastatin	Contraindicated in many cases	Statins are hepatically cleared; monitor LFTs. Atorvastatin and pravastatin are relatively safer.
Ezetimibe	Hepatic via glucuronidation	No adjustment, but monitor LFTs	Use with caution	Not recommended	Increased exposure in hepatic impairment; avoid in moderate–severe cases.
PCSK9 Inhibitors (alirocumab, evolocumab)	Not hepatically metabolized (catabolized like proteins)	No adjustment	No adjustment	Limited data, but generally safe	Safe due to non-hepatic metabolism; monitor for immune reactions.
Inclisiran	Minimal hepatic metabolism (ASGPR-mediated hepatic uptake)	No adjustment	No adjustment, but monitor	Not recommended	Lacks data in Child-Pugh C; generally well-tolerated in mild/moderate impairment.
Bempedoic Acid	Prodrug activated in liver via ACSVL1	Use with caution	Not recommended	Not recommended	Limited data; avoid in advanced liver disease due to unknown risk.
Lomitapide	Extensive CYP3A4 metabolism	Start at low dose	Contraindicated	Contraindicated	High hepatotoxicity risk; black box warning for liver injury. Monitor transaminases and hepatic fat.

Note: Statins: generally contraindicated in severe liver disease, with dose limitations in moderate impairment. Ezetimibe and bempedoic acid: not recommended in moderate-to-severe hepatic impairment due to increased drug exposure. PCSK9 inhibitors and inclisiran: safe and do not require adjustments, but inclisiran lacks safety data in Child-Pugh C. Lomitapide: carries a boxed warning for liver toxicity; contraindicated in moderate-to-severe hepatic impairment.
